# Evaluation of Analytes Characterized with Potential Protective Action after Rat Exposure to Lead

**DOI:** 10.3390/molecules26082163

**Published:** 2021-04-09

**Authors:** Ivan Liakh, Darya Harshkova, Anastasiya Pauliukavets, Vladimir Sheibak, Tomasz Bączek, Natalia Miękus

**Affiliations:** 1 Department of Toxicology, Medical University of Gdańsk, Hallera 107, 80-416 Gdańsk, Poland; liakh_ivan@mail.ru; 2 Department of Plant Physiology and Biotechnology, Faculty of Biology, University of Gdańsk, Wita Stwosza 59, 80-308 Gdańsk, Poland; darya.harshkova@phdstud.ug.edu.pl; 3 S.I. Gelberg Department of Microbiology, Virology and Immunology, Grodno State Medical University, Vilenskaja str., 19, 230-023 Grodno, Belarus; anastasiayk@mail.ru (A.P.); vsheibak@gmail.com (V.S.); 4 Department of Pharmaceutical Chemistry, Faculty of Pharmacy, Medical University of Gdansk, Hallera 107, 80-416 Gdańsk, Poland; tomasz.baczek@gumed.edu.pl

**Keywords:** biological sample preparation, lead intoxication, liquid chromatography, protective agents

## Abstract

Lead (Pb) was revealed for its role as a neurodevelopmental toxin. The determination of neurotransmitters (NTs) in particular brain regions could ameliorate the precise description and optimization of therapeutic protocols able to restore the harmony of signaling pathways in nervous and immune systems. The determination of selected analytes from the group of NTs based on the liquid chromatography (LC)-based method was carried out to illustrate the changes of amino acid (AA) and biogenic amine (BA) profiles observed in chosen immune and nervous systems rat tissues after Pb intoxication. Also, a protective combination of AA was proposed to correct the changes caused by Pb intoxication. After the administration of Pb, changes were observed in all organs studied and were characterized by a fluctuation of NT concentrations in immune and nervous systems (hypothalamus samples). Using a protective mixture of bioactive compounds prevented numerous changes in the balance of NT. The combined analysis of the immune and nervous system while the normalizing effect of curative agents on the level of differentially secreted NTs and AA is studied could present a new approach to the harmonization of those two essential systems after Pb intoxication.

## 1. Introduction

Human exposure to lead—an extremely poisonous heavy metal—could occur with the intake of contaminated food as well as water or through the inhalation of contaminated air. Even though most countries decreased its usage, we could still find it in industries such as car repair, battery storage, recycling, smelting, contaminated pottery in kitchens, and tobacco smoke. European Chemicals Agency (ECHA) reports indicate no observed effect concentration (NOEC) values between 64–1000 mg/kg in diet. US occupational legislation indicates values of 0.050 mg/m^3^ for eight-hour exposures. A safe limit of exposure to lead does not exist [1]. This most common toxic metal causes dose-dependent damage in all human organs, especially in the central nervous system (CNS) of children, but also adults [2,3]. The perturbation caused by lead involves multiple neurotransmitter systems [1,4,5]. That is why dose- and time-dependent exposure to lead could contribute not only to the development of various neurological and neurodegenerative disorders such as Alzheimer’s and Parkinson’s diseases, schizophrenia, behavioral problems, but also affect intellectual functioning and memory. The influence of acute and chronic exposure to Pb was also confirmed to have an impact on the immune system in multiple in vivo and in vitro studies. Some studies revealed that Pb impacts the level of immunoglobulins and lymphocytes, dysregulates antioxidant defenses, and induces oxidative tissue damage [6,7]. Bilateral interactions between the immune and nervous systems were studying deeply for a long time. The nervous system modulates not only immune activity through parasympathetic and sympathetic inputs, but also body temperature, eating, sleep, and, in that way, regulates the nervous system. Also, immune cells—lymphocytes—are present in the brain, where they regulate cognition and take part in the creation of neuronal circuits [8]. Since the CNS and immune system are highly complex, organized, and regulate the entire body, Pb influence on these two major systems should be explored in detail.

The protective effect of zinc on the hematopoietic system, which is most sensitive to the effects of Pb, has been known for long. With an increase in dietary zinc intake, overall toxicity of Pb cations decreases. There is a decrease in Pb accumulation in the blood, liver, and kidneys, a decrease in aminolevulinic acid (ALA) excretion by the kidneys and inhibition of renal ALA-dehydratase activity in rats [9]. Zinc application prevents the inhibition of porphobilinogen-synthase and decreases acetylcholinesterase activity in the brain, and increases Pb accumulation in the blood and brain [3]. Also the use of zinc in the background of the introduction of Pb has a beneficial effect on the reproductive system [10].

The use of tryptophan (Trp) in Pb intoxication is due to the fact that, being a precursor of serotonin, it can help maintain normal levels of components of the serotoninergic system, which is very susceptible to Pb [11,12]. On the other hand, Pb acetate alters the binding affinity of Trp with liver nuclei, which affects protein synthesis in the liver. In addition, recently, the effect of Pb on the kynurenine pathway of Trp metabolism has been shown, which may also contribute to neurocognitive impairment caused by Pb [13].

Taurine (Tau) is a trophic factor in brain development and optimizes neuronal function. This compound protects brain cells from excitotoxicity caused by excitatory AA (glutamate, Glu; aspartate, Asp) in the hippocampus and cerebellum [14]. Tau also prevents the development of an unfavorable metabolic cascade induced by ischemia and hypoxia. It has been shown that high content of Tau in nerve cells makes them more resistant to ischemia and inhibits the development of convulsive reactions [15].

The neuroprotective properties of the inhibitory amino acid Tau in Pb intoxication are presented in a few studies. It has been shown that the use of Tau in Pb intoxication prevents a decrease in glutathione levels, reduces the concentration of malondialdehyde and the activity of catalase and glucose-6-phosphate dehydrogenase [16]. Administration of Tau to rat pups exposed to Pb in the prenatal, perinatal, and lactation periods of development reduces Pb content in the hippocampus, increases the amplitude of long-term potentiation, and can prevent the development of synaptic plasticity deficit in adults [17]. Tau protects against Pb-induced long-term potentiation deficiency in the dentate gyrus of rats, improving synaptic plasticity and cognitive development in offspring [18]. The use of Tau in chronic Pb intoxication increases the activity of superoxide dismutase in the blood and brain of animals and enhances the effect of 2,3-dimercaptosuccinate, which, ultimately, is realized in a decrease in the level of Pb in the blood, liver, and brain [19]. Tau prevented the development of alterations in neurochemical profiles in areas of the brain [20], and also improved the results of labyrinth tests when exposed to Pb [21].

There is a long-known ability of arginine (Arg) as a potent source of nitric oxide to lower blood pressure when added to a diet [22]. Pb-induced hypertension is also amenable to correction with Arg. Moreover, the use of L-arginine has a positive effect on learning, brain damage, and memory impairment due to oxidative stress [23]. Even a high level of consumption of Arg and Tau is safe and has no observable adverse effect [24]. In addition, studies of the distribution of ^14^C Arg and ^14^C Tau in organs showed that Arg has an accumulation pattern in the spleen, and Tau in the brain, the organs which were the subject of our study. The purpose of this experiment was to find out whether it is possible to use a mixture of the above-mentioned AA in combination with zinc aspartate to reduce Pb-induced changes in the nervous and immune systems.

## 2. Results

The study included the LC-based analysis of the selected compounds evaluated in hypothalamus, liver lymphocytes, liver, spleen lymphocytes, spleen, and plasma. After the experiment, samples of the striatum and midbrain were also taken to analyze the levels of neurotransmitters. In the striatum, the administration of lead acetate was accompanied by an increase in the level of glutamate (18%) and citrulline (19%), which normalized upon administration of the AAmix. On the contrary, there was a decrease in the levels of threonine (38%), arginine (35%), citrulline, and ethanolamine (22%) in the midbrain. Except for the excitatory amino acid glutamate in striatum, the neurotransmitter systems have not changed in these brain structures, in contrast to the hypothalamus results which are described in this article. In this study, the hypothalamus was of greater interest in comparison with other structures of the brain, since, together with the most important neuroregulatory functions of the hypothalamus, the nervous and humoral systems intersect in it. In addition, due to the structural features of the blood–brain barrier, the hypothalamus most quickly and noticeably reacts to changes biochemical parameters in the blood.

### 2.1. The Changes Observed in the Brain

**Figure 1:**
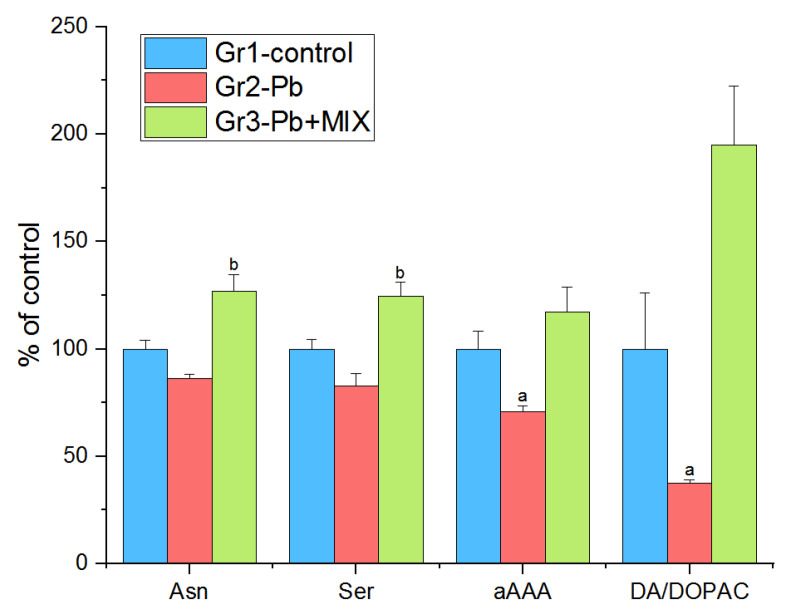
The changes of the biomarkers evaluated in hypothalamus (*n* = 6; a indicates statistically significant differences between control and Group 2; b indicates statistically significant differences between Group 3 and Group 2). The results correspond to the mean values (±SE), *p* < 0.05.

Our study of the level of neurotransmitters in the brain structures of adult rats on day 11 after intragastric administration of Pb acetate (75 mg/kg) on the first and fifth day of the experiment showed the following changes: in the hypothalamus, the strongest statistically significant increase was noted in Group 3 vs. Group 2 for the levels of asparagine (Asn) (*p* < 4 × 10^−3^) and serine (Ser) (*p* < 0.03). Also, the levels of α-aminoadipic acid (aAAA) (*p* < 0.02) and DA turnover—dopamine/3,4-dihydroxyphenylacetic acid ratio (DA/DOPAC)—(*p* < 0.03) in Group 2 were significantly decreased by 15–30% as compared to those in the control group. Therefore, the administration of the AAmix eliminated the change levels of aAAA (Figure 1).

### 2.2. The Changes Observed in Peripherial Tissues

**Figure 2:**
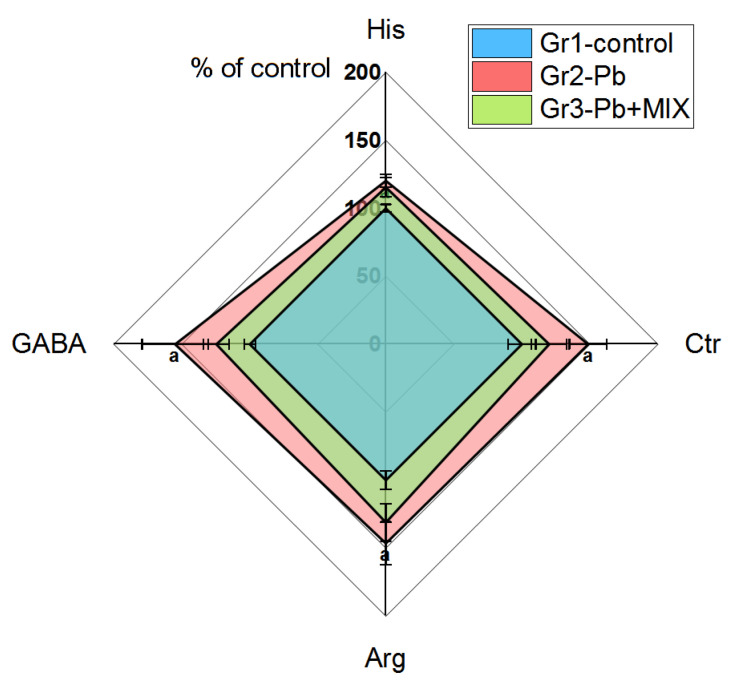
The changes of the biomarkers evaluated in liver (*n* = 6; a indicates statistically significant differences between control and Group 2. The results correspond to the mean values (±SE), *p* < 0.05.

In liver tissue, the levels of histidine (His) (*p* < 0.02), citrulline (Ctr) (*p* < 0.03), Arg (*p* < 0.04), and gamma-aminobutyric acid (GABA) (*p* < 0.05) in Group 2 were increased by about 20–55% as compared to those of the control (Figure 2). Moreover, in liver lymphocytes, even more changes could be seen: the levels of Asn (*p* < 0.025), threonine (Thr) (*p* < 0.01), Tyr (*p* < 0.03), and valine (Val) (*p* < 0.04) were significant decreased by 30–50% as compared to those of the control group; however, the level of glutamine (Gln) (*p* < 0.035) in Group 2 vs. Group 3 was increased (Table 1). Summarizing the above, the addition of AAmix normalized all biomarkers in liver tissue and its lymphocytes, except the level of Gln.

Based on our results, we also noted changes in spleen tissue after administration of Pb acetate such as decreased levels of β-aminobutyric acid (bABA) (*p* < 0.02) by about 50% when compared to that in the control group.

**Figure 3:**
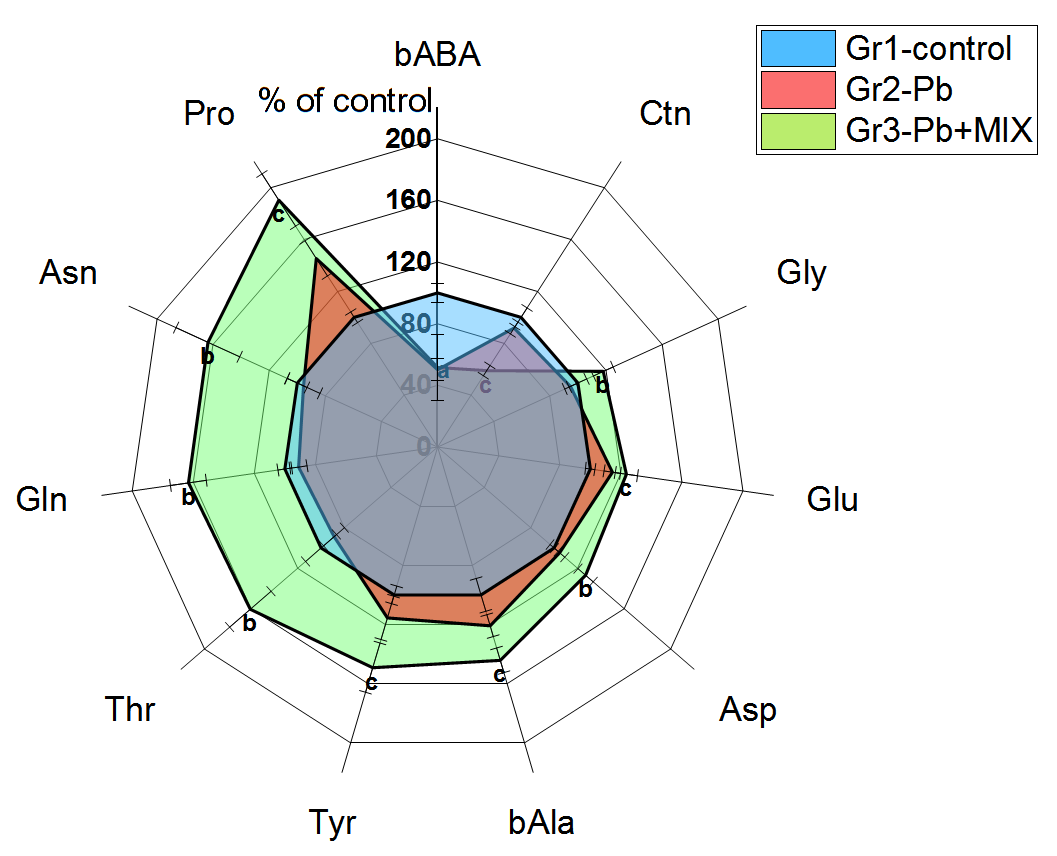
The changes of the biomarkers evaluated in spleen (*n* = 6; a indicates statistically significant differences between control and Group 2; b indicates statistically significant differences between Group 3 and Group 2; c indicates statistically significant differences between Group 3 and the control group). The results correspond to the mean values (±SE), *p* < 0.05.

What is more, statistically significant increases were noted in Group 3 vs. Group 2 for the levels of Asp (*p* < 0.02), Asn (*p* < 0.01), Gln (*p* < 0.0002), glycine (Gly) (*p* < 0.05), and Thr (*p* < 0.007). Additionally, we observed that the levels of Glu (*p* < 0.02), β-alanine (bAla) (*p* < 0.02), Tyr (*p* < 0.05), Ctn (*p* < 0.0007), and proline (Pro) (*p* < 0.0007) in Group 3 were higher than those in the control group. We also noted changes in some indicators of amino acid metabolism; statistically significant increases were noted in Group 3 vs. Group 2 for nonessential AA (*p* < 0.02), proteinogenic AA (*p* < 0.03) by 23% and 25%, respectively, and a statistically significant decrease by 40% in Glu/Gln ratio (*p* < 0.0002). Total contents of AA (*p* < 0.01) and aromatic AA (*p* < 0.003) in Group 3 were significantly increased by 20% and 73%, respectively, compared to those in the control group. (Figure 3).

In spleen lymphocytes in Group 2, the levels of Arg (*p* < 0.009) and Pro (*p* < 0.03) were decreased by 30–60% compared to those in the control group. On the other hand, the levels of His (*p* < 0.02) and Gly (*p* < 0.03) in Group 3 were almost two times higher compared to those of Group 2. The treatment with AAmix brought normalization of the level of Arg and Pro in spleen lymphocytes, however, in spleen tissue, the biomarkers did not reach the control value (Table 1).

**Figure 4:**
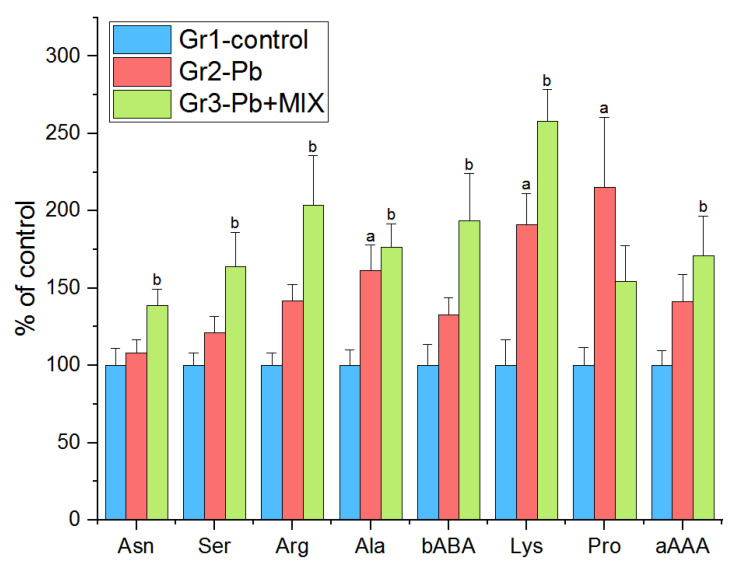
Changes of biomarkers evaluated in plasma (*n* = 6; a indicates statistically significant differences between control and Group 2; b indicates statistically significant differences between Group 3 and control group). The results correspond to the mean values (±SE), *p* < 0.05.

The changes of plasma reflected the effects of acute Pb intoxication: most of the parameters tested in Group 2 were increased (the level of alanine (Ala) (*p* < 0.01), lysine (Lys) (*p* < 0.006), and Pro (*p* < 0.03)) as compared to those of the control group. The levels of Asn (*p* < 0.05), Ser (*p* < 0.01), Arg (*p* < 0.002), aAAA (*p* < 0.03), and bABA (*p* < 0.005) in Group 3 were also higher when compared to those of the control group. Unfortunately, correction with the AAmix normalized only the level of Pro in plasma (Figure 4).

## 3. Discussion

**Figure 5:**
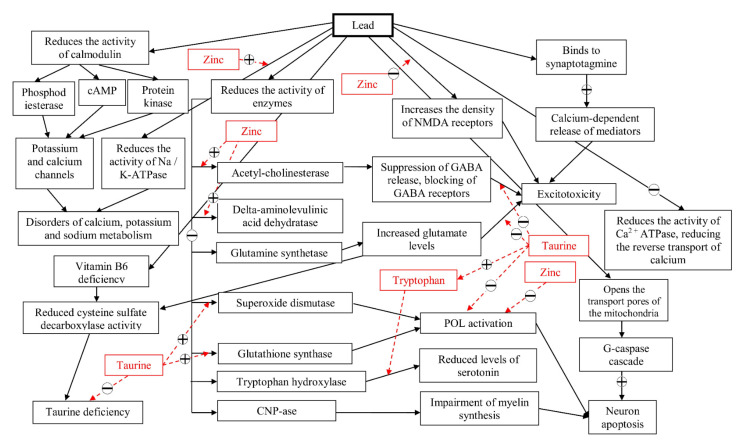
The main molecular mechanisms of the deteriorating effect of lead on nervous tissue and the ways in which the components of the mixture influence these mechanisms. Note: “+”—parameter gain; “−”—parameter reduction.

What unites the nervous and immune systems is that in both cases, their functioning occurs due to the release of chemical signals (cytokines and mediators) that regulate the behavior of individual cells, lymphocytes and neurons, respectively. In addition, interactions at the level of direct contacts (receptor–ligand interaction in the case of immune cells and synapses in the case of nerve cells) play an important role in these systems. Logically following from the above, the similarity of the nervous and immune systems is their strong dependence on the metabolism of mediators, which, in turn, is closely intertwined with the metabolism of amino acids and biogenic amines. Thus, the effects of lead on amino acid and neurotransmitter balance of the whole organism can have a great impact on the nervous and immune systems, at the same time making it difficult to understand the local effect of lead on the cells and organs of these systems. LC-based approaches are among great laboratory tools to study the complex relations between organism intoxication and endogenous substance disturbances. Still, knowing the complex mechanism by which Pb influences the levels of individual AAs, the results obtained in presented research should be considered carefully after description of Pb potential mechanisms of actions towards a particular AA. Pb might have an impact on the synthesis and degradation of amino acids by acting on the corresponding enzymes (direct damage to proteins or by replacing metal ions in active centers). Pb can also directly bind to amino acid groups (thiol-, hydroxyl-, amine-, and carboxyl groups). As a consequence, AA binding with transport proteins is modified, affecting intra- and extracellular amino acid flows. Moreover, a mediator AA cannot bind to the receptors, and therefore their signaling function is impaired. Also, the serious impact of Pb on AAs could cause major complications in protein synthesis (see Figure 5). It is even more difficult to assess the consequences of the damaging effect of Pb based on changes in the levels of individual AAs, since they have various effects in particular organs. Below, the possible explanation and consequences of obtained data are discussed.

### 3.1. Hypothalamus

Dopaminergic dysfunction in the brain upon exposure to Pb is well-documented and associated with multiple learning and behavioral disorders [25,26]. Literature data indicates that the increased activity of neurotransmitter systems, which is observed during Pb intoxication and is responsible for the development of depression, is associated with damage to neurons as a result of the formation of large amounts of free radicals, which occurs in response to decreased activities of antioxidant enzymes [27]. Other studies suggested that dopaminergic effects are an indirect result of the effects of Pb on the glutamatergic and cholinergic systems [28]. An increase in DA metabolite levels in the hypothalamus, as observed in the present study (Figure 1), may be associated with an increase in DA catabolism due to its oxidative deamination by monoamine oxidase, observed after exposure to Pb [26]. On the other hand, a decrease in DA levels may be due to inhibition of enzymes of its synthesis [29], and also possibly due to the formation of a complex of DA with Pb, as it happens with iron intoxication [30]. However, these changes are difficult to describe by one unified mechanism, since the action of Pb on the dopaminergic system in different regions of the brain is significantly different, as the obtained results indicated (data not shown), which is associated with differences in the processes of synthesis, utilization, and reuptake of DA, as well as the ratio and number of DA receptors. Regardless of the mechanism involved, the use of the AAmix prevented changes in dopamine metabolism in the hypothalamus caused by lead.

Serine plays an important role in the CNS; Ser is a precursor of the neurotransmitter Gly. Additionally, D-serine, synthesized from L-serine by serine racemase, is an activator of NMDA receptors in the brain [31]. Serine has been shown to reduce oxidative stress and inflammation that occur in the hypothalamus [32]. Changes in Ser levels can be directly related to its involvement in the metabolism of a one-carbon unit, or due to the increased conversion of Ser to Gly and cysteine (Cys), which are required for the synthesis of glutathione to protect against Pb-induced oxidative stress. The observed increased Ser content in Group 3 in comparison with those of Group 2 may indicate a weakening of Ser utilization in the processes described above and the restoration of normal protective functions of Ser in the hypothalamus.

Recently, it has been shown that synthesis of Asn is necessary for the development and functioning of the brain, and depletion of Asn reserves can lead to neurological disorders [33]. Despite the fact that food intake may be a source of Asn to the brain, a more plausible reason for the decrease in its level in Group 2 compared to that of Group 3 was a decrease in endogenous synthesis by asparagine synthetase (an enzyme that is very sensitive to external influences [34]) caused by lead or, accordingly, the activating effect of the components of the AAmix on this enzyme.

L-α-aminoadipic acid is a natural metabolic product of lysine with neuroexcitatory properties, being a structural homologue of the excitatory amino acid Glu, aAAA can affect various elements of glutamatergic neurotransmission, moreover, it inhibits the production of the neuroinhibitory metabolite kynurenic acid in the brain [35,36]. A possible decrease in the level of aAAA in Group 2 was due to the fact that a large part of lysine is metabolized to form pipecolic acid [37]. The use of AAmix prevented the change in aAAA levels.

### 3.2. Liver Tissue and Liver Lymphocytes

Metabolism of AAs in the liver is very active, since the liver is the main site of protein synthesis; the processes of transamination, oxidation of AA in the citrate cycle, and their use for gluconeogenesis are actively taking place in the liver. AAs are not only nutrients, they, as well as their metabolites formed in the liver, are responsible for a huge number of biological functions [38,39]. Arg is a very important amino acid in the implementation of cellular immune functions (synthesis of protein, NO, creatine, urea) [40]. An increase in Arg and Ctr in Group 2 may be associated with a functional block in the urea cycle, which may result from Pb-induced hepatorenal dysfunction [41,42]. Interestingly, despite the fact that the AA-mix contained Arg, the levels of Arg and Ctr were lower in the third group of animals than those in the second group and did not differ from those of the control, which may indicate that the AAmix led to activation of the processes of using these AA.

Outside the CNS, GABA synthesis is weak; one of the sources of GABA is synthesis by bacteria in the intestine, which is then metabolized in the liver [43]. Due to the low activity of glutamate decarboxylase, the liver is not an active producer of GABA [44], however, GABA-ergic signaling in hepatocytes plays an important role in the reduction of toxin-induced apoptosis [45,46]. The increased GABA level in Group 2 may be due to a decrease in its conversion to succinate for use in the Krebs cycle for energy production [47]. The positive effect of AAmix on this parameter can be explained by the use of its components as more accessible energy substrates in the Krebs cycle instead of GABA.

An increase in the level of His in Group 2 may be associated with a weakening of its catabolism due to a decrease in the activities of enzymes involved in its transformations in the liver, such as histidase, urocanase, and formimino transferase, with the latter enzyme being highly dependent on the level of tetrahydrofolate, which, in turn, is associated with the S-adenosylmethionine/S-adenosylhomocysteine system [48], which are known to be highly responsive to Pb exposure [49]. Disruption of the enzymatic systems of histidine degradation is also indicated by the fact that these animals do not exhibit changes in the concentrations of Glu, Ala, Gln, and branched-chain amino acids (BCAA), which is normally observed with a high level of His and its increased flux through the His degradation pathway [48].

Normal levels of Asn in lymphocytes are essential for normal functioning of the immune system. Despite the fact that Asn can be synthesized in lymphocytes [50], with a decrease in the level of its precursor Gln (which was also observed in Group 2), its deficiency may occur [51], which has an immunosuppressive effect [52,53]. In addition, Asn deficiency leads to impaired growth of lymphocytes and activation of their apoptosis, reduced expression of ornithine decarboxylase, and synthesis of polyamines [52]. Gln is no less important for lymphocytes than Asn, but unlike it, it is essential for lymphocyte proliferation. The observed downward trend in Gln levels in Group 2 can be explained by a shift in equilibrium from its synthesis (low activity of glutamine transaminase) to the side of conversion to Glu (by the action of glutaminase, which is normally increased in lymphocytes) and other products (Asp, Asn, Ala, and lactate) [51]. The restoration of the role of glutamine transaminase may explain the increase in Gln levels in Group 3 [51]. Threonine is essential for cell growth and antibody production in lymphocytes [52]. A decrease in the level of Thr in Group 2 may be associated with a decrease in its synthesis in hepatocytes [54]. In addition, a similar decrease is observed in sepsis and is associated with the utilization of Thr for the synthesis of acute phase proteins, intestinal proteins, and mucins [55]. From this point of view, the restoration of the threonine level in Group 3 may indicate a weakening of the above-mentioned negative processes.

Both Val and Tyr levels were markedly reduced in Group 2. Tyr is not an essential amino acid for rats, and its short-term deficiency is tolerated by lymphocytes quite easily [53,56]. However, its importance for lymphocytes has recently been overestimated, since it was found that adrenergic receptors are expressed on the surface of lymphocytes, the activation of which by tyrosine-derived catecholamines stimulates the differentiation and proliferation of Th1 cells and B cells [57]. Because the carbon skeletons of BCAA are not synthesized in lymphocytes, Val, like Tyr, is essential for lymphocyte proliferation [53]. Considering that Val is an essential amino acid and all the enzymes involved in the Val oxidation pathway are expressed in lymphocytes, a decrease in its level indicates an increase in its catabolism [58] which can be a compensation for the altered glucose metabolism [59].

### 3.3. Spleen and Spleen Lymphocytes

Significant changes in amino acid levels in the spleen in Group 3 can be explained by a high level of correlation of venous AA with splenic AA [60], while the spleen can contribute 10% or more of the splanchnic extraction of AA [61]. This is supported by the fact that, as in plasma, most of the changes in amino acid levels were also upward (Figure 2). In Group 2, there was only a decrease in the level of bABA—a nonprotein amino acid end product of pyrimidine metabolism (thymine catabolite). So, a decrease in the level of bABA may be associated with a decrease in the utilization of thymine of erythroblast origin [62], although these data are not entirely consistent with the inhibitory effect of Pb on the hematopoietic system [63], which can be explained by the shorter exposure time to Pb in comparison with that of other studies. It can be assumed that after one week of exposure to Pb, there is still no mass destruction of erythrocytes in the spleen, but at the same time, the total number of erythrocytes decreases and, consequently, their decay products also decrease [64,65].

Given the high content of histamine in macrophages and lymphocytes of the spleen [66], the lower level of His (a precursor of histamine) in Group 2 vs. Group 3 may be associated with the induction of histidine decarboxylase by Pb [67], and may significantly modulate immune responses [68]. Being a source of NO—a signaling molecule responsible for many immunological functions, Arg is an important amino acid for lymphocytes [52], and Arg deficiency observed in Group 2 can inhibit lymphocyte proliferation [53]. Normalization of the level of Arg in Group 3 of animals may be explained by its additional intake with AAmix. The decrease in Pro level observed in Group 2 may be associated with its enhanced oxidation by proline oxidase through the proline cycle to cover the energy needs of lymphocytes under conditions of oxidative stress [69,70]. Hydrogen peroxide formed in the proline cycle is involved in the regulation of the redox state, which affects the growth, proliferation, and apoptosis of lymphocytes, as well as the production of antibodies [52].

### 3.4. Plasma

It is especially difficult to assess the consequences of the damaging effect of Pb in terms of changes in the levels of individual AAs in plasma, since their levels and composition are subject to rapid changes during digestion and depend on food composition [71], however, their changes can be associated with the level of plasma proteins [72] and even reflect a violation of the synthesis of neurotransmitters in the brain [73,74]. However, a persistently elevated level of a particular amino acid may indicate impaired breakdown (metabolism) of that amino acid.

Based on the functions of Ala, its high level in Group 2 of animals may indicate a weakening of the glucose-alanine cycle, which may be due to both a decrease in gluconeogenesis and weakening of urea synthesis in the liver [41,75]. A higher level of Pro observed in Group 2 may indicate its poor utilization, which may be associated with a weakening of its transformation into collagen due to a deficiency of vitamin C and iron in Pb poisoning [76,77], as well as a decrease in the level of niacin, a cofactor of proline oxidation to glutamate [78]. The same reason (decreased levels of Vitamin C, niacin, B6, and iron) can explain the violation of lysine metabolism and an increase in its level [79]. Also, with suppression of Lys metabolism, vitamin B6 deficiency and dysfunction of pyridoxal-5-phosphate as a coenzyme for transamination may explain the accumulation of aAAA—an intermediate in the metabolism of Lys [80]. An increase in the level of Arg in plasma in Group 3 may be due to its presence in the AAmix, as well as a weakening of its conversion to Ctr by enterocytes, a less possible reason may be the increased conversion of Ctr back to Arg in the kidneys, since the level of Ctr did not change in plasma and other organs in this group of animals [81].

## 4. Materials and Methods

### 4.1. Reagents

Lead acetate (Pb(C_2_H_3_O_2_)_2_3H_2_O), molecular weight (MW) 379.33, with laboratory reagent grade was obtained from the Department of General and Bioorganic Chemistry of Grodno State Medical University (Grodno, Belarus). A mixture of bioactive molecules for treatment (AAmix) was prepared from L-arginine (Carl Roth, Karlsruhe, Germany), L-taurine (Sigma-Aldrich, Tokyo, Japan), L-tryptophan (Carl Roth, Karlsruhe, Germany), zinc aspartate (Sigma-Aldrich, Seelze, Germany) in a molar ratio of 4:4:1:1 diluted in water. The mixture we used is known in Russian scientific literature under the name “tritarg”. The mixture was developed at the Grodno State Medical University and is well-described in Russian scientific literature. In a series of in vitro and in vivo experiments, it was found that the AAmix has a pronounced immunomodulatory effect in various target organs [82,83,84]. Further studies have shown the effectiveness of AAmix in experimental insufficiency of tryptophan and for metabolic correction of alcohol intoxication [85] (patent “Means for correcting liver dysfunctions in intermittent alcohol intoxication”; No.19802 from 28 February 2016 [86]). The above properties of this composition were taken into account when choosing it for use in our study. Ultra-pure water was prepared from distilled water using a Milli-Q system (Millipore, Bedford, MA, USA). Other reagents (acetic acid, sodium acetate, perchloric acid, chlorine acid, 5-aminovaleric acid, EDTA, sodium metabisulfite, o-phthalaldehyde, 3-mercaptopropionic acid, sodium heptyl sulfonate, sodium octyl sulfonate, potassium dihydrogen phosphate, borax, sodium hydroxide, methanol, urographin, and standards for AA and BA) were of the highest quality available and were obtained from commercial sources.

### 4.2. Animal Care and Use

Animal experimental procedures were approved by the Biomedical Ethical Committee of the Grodno State Medical University and performed while following the established legal guidelines of the European Convention for the Protection of Vertebrate Animals used for Experimental and Other Scientific Purposes (1986, ETS 123). The experiment was carried out in accordance with the rules and norms of bioethical treatment of experimental animals (Order of the Ministry of Health of the Republic of Belarus No.274 of 04/17/2006).

### 4.3. Animal Models and Experimental Procedure

The study was performed using 18 heterogeneous white male rats weighing 150–200 g, which were placed with free access to water and fed standard food. The animals were divided into three groups: (1) control group (*n* = 6); (2) research group to which Pb acetate (75 mg/kg) was administered intragastrically on the first and fifth day of the experiment (total = 1/30 of LD50) (*n* = 6); (3) research group to which Pb acetate (75 mg/kg) was administered intragastrically on the first and fifth day of the experiment, and AAmix (300 mg/kg) was administered intragastrically (*n* = 6). On the 11th day of the experiment, all the rats were decapitated. The brain was immediately removed, the hypothalamus was dissected on ice and stored in liquid nitrogen for later analysis [87]. Liver and spleen samples (50–100 mg of tissue) were also taken into Eppendorf tubes as well as plasma samples (in sterilized EDTA probes). The samples were kept in −80 °C until the analysis.

### 4.4. Isolation of Liver and Spleen Lymphocytes

Lymphocytes were isolated in the density gradient of urographin [88]. The method consists in the fact that when centrifuging after layering on a gradient, it is possible to separate cells having a density lower (lymphocytes, monocytes) and higher (red blood cells, granulocytes) than 1.077 g/mL. Urographin was diluted with distilled water to a density of 1.077 g/mL. Blood was collected in heparin tubes (25 units in 1 mL of blood). Liver tissue was crushed with scissors and then thoroughly triturated in a homogenizer in buffered saline. Blood was diluted two times, and tissue homogenates in a volume of 4 mL were layered on urographin (2 mL), followed by centrifugation (4000 rpm, 5 min, 4 °C). During centrifugation, red blood cells and granulocytes settled to the bottom of the tube. On the upper boundary of the gradient, with proper separation, a loose whitish ring was formed, consisting mainly of lymphocytes. Plasma was located above the lymphocyte layer. Lymphocytes were collected in a dry conical centrifuge tube. Two to three milliliters of physiological saline was added to the suspension of lymphocytes, thoroughly mixed, and centrifuged at 1500 rpm, 5–7 min, 4 °C. The supernatant was removed, and the washing procedure was repeated one more time. Then, the concentration of lymphocytes in the Goryaev chamber was calculated. To calculate the concentration, 50 μL of lymphocyte suspension was taken, and 1 mL of 3% acetic acid was added. In the Goryaev chamber, lymphocytes were counted in 100 large squares; the obtained number was multiplied by 50,000. The result corresponded to the number of lymphocytes in 1 mL of suspension.

### 4.5. Analysis of the Concentrations of Chosen Amino Acids and Biogenic Amines

The determination of the levels of AA was carried out in perchloric acid extracts as previously described [89]. A tissue sample (20–80 mg) was weighed and homogenized in 10 volumes of 0.2 M perchloric acid containing an internal standard (400 nM 5-aminovaleric acid (dAVA)), and 50 mg/L of EDTA and 50 mg/L of sodium metabisulfite as an antioxidant to preserve the analytes from decomposition were added. To obtain plasma, whole blood was collected in sterile glass tubes containing heparin and centrifuged (4000 rpm; 4 °C; 10 min). A similar extraction procedure was used for plasma, except that the plasma was mixed with one volume of perchloric acid. The resulting homogenates and plasma precipitate were centrifuged (12,000 rpm, 15 min, 4 °C), and the supernatant was immediately separated from the precipitate. AAs were then derivatized with o-phthalaldehyde and 3-mercaptopropionic acid, separated and determined with the use of reverse phase high-performance liquid chromatography (RP-HPLC, Agilent 1100 chromatographic system (Agilent technologies, Santa Clara, CA, USA)) with isocratic elution and fluorescence detector (231/445 nm). Detailed procedures are presented below.

HPLC conditions: Zorbax Eclipse Plus C18 column 2.1 × 150 mm, 3.5 µm; the mobile phase consisted of 0.1 M Na-acetate buffer pH 5.7 mixed with 50% methanol in a ratio of 100/54 (*v*/*v*), 12.38 g of sodium acetate and 0.81 g of acetic acid were added to 800 mL of distilled water, the solution was brought to the final desired pH value using HCl or NaOH, distilled water was added to a volume of 1 L, and the resulting solution was mixed with methanol. Flow rate was 0.8 mL/min, and column temperature was 30 °C.

Derivatization: mixing the sample with 5 volumes of a 0.4% solution of o-phthalaldehyde and 0.3% 3-mercaptopropionic acid in 0.4 M Na-borate buffer, pH 9.4 (38.14 g of Borax 1 and 3.52 g of sodium hydroxide were added to 800 mL of distilled water, the solution was brought to the final desired pH value using HCl or NaOH, distilled water was added to a volume of 1 L), followed by neutralization by adding an equal volume of 0.1 M hydrochloric acid.

Homogenization and preliminary sample preparation for studying the levels of BA did not differ from tissue sample preparation for determining the levels of neuroactive AA. Further, the HLPC method was modified; for BA, their precursor and metabolite determination, the ion-pair HPLC method was used (Agilent 1200 chromatographic system (Agilent technologies, Santa Clara, CA, USA)). The conditions are presented below.

HPLC conditions: Zorbax Eclipse Plus C18 column (2.1 × 150 mm), 3.5 µm; mobile phase: 0.1 M potassium dihydrogen phosphate buffer pH 3.55 (13.6 g of potassium dihydrogen phosphate, 0.8 mL of acetic acid, 200 mg of sodium heptyl sulfonate, 200 mg of sodium octyl sulfonate, and 30 mg of EDTA were added to 800 mL of distilled water, the solution was brought to the final desired pH value using HCl or NaOH, distilled water was added to a volume of 1 L, and the resulting solution was mixed with methanol (11.5 vol.%). Flow rate was 0.5 mL/min, column temperature was 27 °C. Detection by fluorescence, excitation wavelengths of 280 nm, emission 320 nm (for determining tyrosine (Tyr) and its derivatives) and 340 nm (for determining Trp and its derivatives), time constant 2 s. The determinations were carried out using Agilent 1100 and Agilent 1200 chromatographic systems (Agilent technologies, Santa Clara, CA, USA); data were received and processed using Agilent ChemStation A10.01 software (Agilent Technologies, Waldbronn, Germany). Examples of chromatograms of free amino acids and biogenic amines in the studied organs of rats are in Supplementary Materials (Supplementary Figures S1–S14).

### 4.6. Statistical Analysis

All statistical analyses were conducted using STATISTICA 10.0 software (StatSoft, Krakow, Poland), all values are presented as mean ± standard deviation (SD). Finally, a parametric test for more than two independent samples of different sample sizes was applied, namely one-way analysis of variance (ANOVA). First, the opportunity to perform parametric test was checked, assuring that all three main assumptions were fulfilled: normal distribution—checked with Shapiro–Wilk test and Kolmogorov–Smirnov test, homoscedasticity (homogeneity of variance)—checked with Levene’s test and/or Brown–Forsythe test, and the correlation of means and variances. The significance of intergroup differences was verified with significance level, *p* < 0.05 (HSD Tukey’s test).

## 5. Conclusions

The LC-based analytical platform applied in the presented study allowed to reveal that even a double administration of Pb in relatively small doses affected the two main systems that regulate the entire body—the CNS and the immune system. In addition to pronounced central and peripheral effects, the metabolism of AAs in lymphocytes underwent significant changes, which was characterized by a decrease in the levels of many AAs, including those essential for vital activity and normal functioning of lymphocytes. The most noticeable quantitative changes in the levels of amino acids lead acetate caused in the liver and liver lymphocytes, the greatest corrective effect of the mixture was also noted there. At the same time, the opposite picture was observed in the spleen, where the most numerous changes occurred with the joint introduction of lead acetate and the mixture.

The presented data proved for the first time that the use of our proposed AAmix, composed of L-arginine, L-taurine, L-tryptophan, and zinc aspartate, prevented numerous changes in the balance of NTs which are relevant to restoring physiological homeostasis in the body. Our findings could lead to further improvements in curing Pb poisoning in humans.

## Figures and Tables

**Table 1:** The changes of the biomarkers evaluated in liver lymphocytes and spleen lymphocytes.

	Gr1-Control	Gr2-Pb	Gr3-Pb + MIX
	Mean ± SD	Mean ± SD	Mean ± SD
Liver lymphocytes, nmol/1 × 10^6^ cells			
Asn	1.77 ± 0.26	0.96 ± 0.09 ^a^	1.69 ± 0.19
Gln	8.11 ± 1.05	4.73 ± 1.38	10.34 ± 1.77 ^b^
Thr	5.18 ± 0.58	2.98 ± 0.18 ^a^	7.71 ± 3.01
Tyr	3.13 ± 0.31	1.62 ± 0.12 ^a^	4.83 ± 1.95
Val	6.60 ± 0.77	3.80 ± 0.25 ^a^	10.68 ± 3.78
Spleen lymphocytes, nmol/1 × 10^6^ cells			
His	2.93 ± 0.29	2.02 ± 0.18	3.50 ± 0.42 ^b^
Gly	20.00 ± 2.07	13.32 ± 1.74	25.33 ± 4.28 ^b^
Arg	4.99 ± 0.57	2.74 ± 0.24 ^a^	4.17 ± 0.76
Pro	36.83 ± 8.96	13.51 ± 1.80 ^a^	21.86 ± 2.49
Nonessen. AA	160.76 ± 18.21	98.05 ± 9.94 ^a^	156.64 ± 24.48

Nonessen. AA: total content of nonessential AA. Data are presented as mean values ± SE (*n* = 6), *p* < 0.05; ^a^ indicates statistically significant differences between control and group 2; ^b^ indicates statistically significant differences between group 3 and group 2.

## Data Availability

Data are available from the authors by request.

## Supplementary Materials

The following are available online, Figure S1: Chromatogram of free amino acids in rat liver, Figure S2: Chromatogram of free amino acids in rat liver lymphocytes, Figure S3: Chromatogram of free amino acids in rat spleen, Figure S4: Chromatogram of free amino acids in rat spleen lymphocytes, Figure S5: Chromatogram of free amino acids in rat plasma, Figure S6: Chromatogram of free amino acids in rat hypothalamus, Figure S7: Chromatogram of biogenic amines in rat liver, Figure S8: Chromatogram of biogenic amines in rat liver lymphocytes, Figure S9: Chromatogram of biogenic amines in rat spleen, Figure S10: Chromatogram of biogenic amines in rat spleen lymphocytes, Figure S11: Chromatogram of biogenic amines in rat hypothalamus, Figure S12: Chromatogram of biogenic amines in rat plasma, Figure S13: Typical chromatogram of a mixture of biogenic amine standards, Figure S14: Typical chromatogram of a mixture of amino acids standards.

## Sample Availability

Samples of the compounds are not available from the authors.
