# Supplementary material for: Evaluation of Analytes Characterized with Potential Protective Action after Rat Exposure to Lead
Source: Molecules. 2021 Apr 9;26(8):2163. doi: 10.3390/molecules26082163 (PMC8069014; doi:10.3390/molecules26082163)
Supplement: Supplementary file 1 [file molecules-26-02163-s001.pdf]

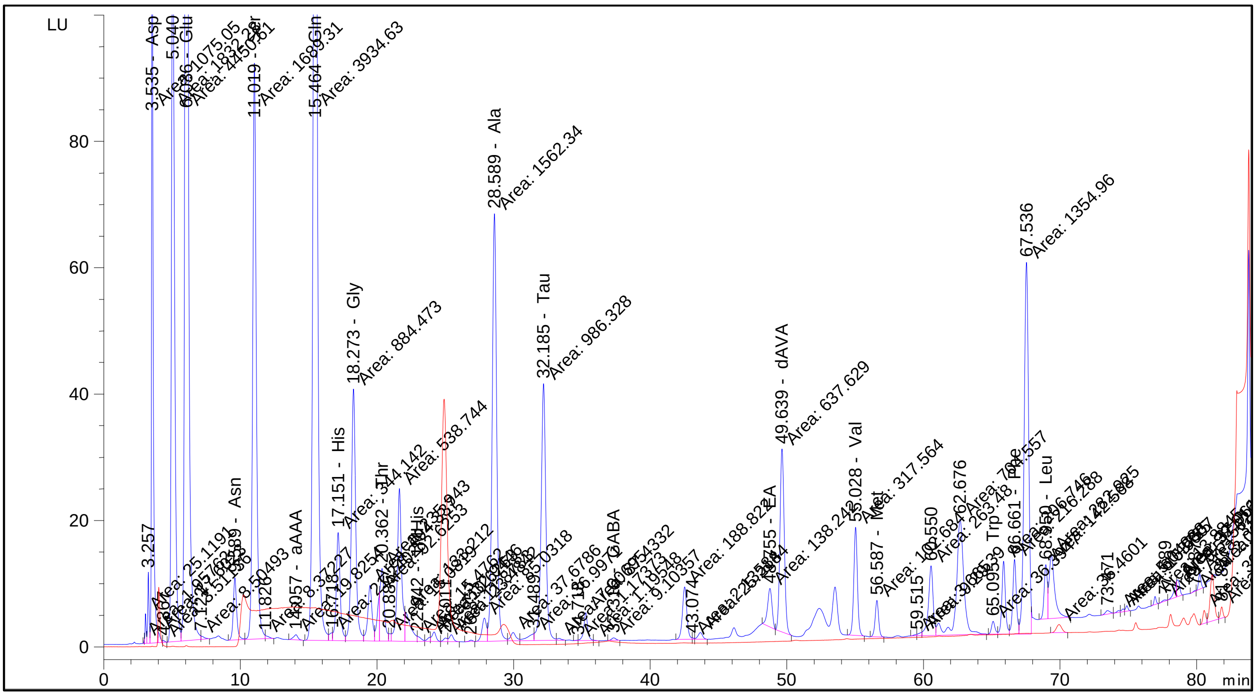

Figure S1. Chromatogram of free amino acids in rat liver.

Note: in this and Supplementary Figures 2-6, 13. – 1Mhis, 1methyl histidine; 3Mhis, 3-methyl-histidine; aAAA, alpha-aminoadipic acid; aABA, alpha-aminobutyric acid; Ala, alanine; Asn, asparagine; Asp, aspartic acid; Arg, arginine; Met, methionine; bABA, beta-aminobutyric acid; Ctr, citrulline; EA, ethanolamine; His, histidine; HPro, hydroxyproline; Ile, isoleucine; GABA, gamma-aminobutyric acid; Gln, glutamine; Glu, glutamic acid; Gly, glycine; Leu, leucine; Lys, lysine; PEA, phosphoethanolamine; Pro, proline; Phe, phenylalanine; Ser, serine; Tau, taurine; Thr, threonine; Trp, tryptophan; Tyr, tyrosine; Val, valine; the blue chromatogram corresponds to the FLD signal at Ex = 231nm and Em = 445nm, and the red one - at Ex = 231nm and Em = 313nm.

```
=====================================================================
                     Figure S1. Internal Standard Report
=====================================================================
Sorted By               :      Retention Time
Multiplier:                     :      1.0000
Dilution:                       :      1.0000
Use Multiplier & Dilution Factor with ISTDs
Sample ISTD Information:
ISTD  ISTD Amount   Name
  #     [nmol/g]
----|-------------|-------------------------
  1      1.00000   dAVA
Signal 1: FLD1 A, Ex=231, Em=445, TT
Signal 2: FLD1 B, Ex=231, Em=313, TT
RetTime Sig Type  ISTD   Area      Amt/Area     Amount   Grp   Name
 [min]            used LU     *s     ratio      [nmol/g]
-------|--|------|----|----------|----------|----------|--|---------------
  3.157  1           1      -          -          -        CA
  3.535  1 MF   +    1 1075.05273 1120.49760 1889.17578    Asp
  6.036  1 FM   +    1 4450.61182  809.26055 5648.58617    Glu
  9.589  1 BV        1  171.24995  837.33521  224.88552    Asn
 11.019  1 MF   +    1 1689.31274  830.09916 2199.23532    Ser
 14.057  1 MF        1   19.82537  866.61774   26.94514    aAAA
 15.464  1 MF        1 3934.63403 1069.26733 6598.15194    Gln
 17.151  1 MF        1  344.14233 1515.15496  817.76181    His
 18.273  1 MF        1  884.47302 1923.29591 2667.85591    Gly
 18.723  1           1      -          -          -        3MHis
 19.477  1 MF        1  194.33658 4571.49098 1393.29824    PEA
 20.362  1 FM        1  235.94275  803.23289  297.22120    Thr
 22.942  1 FM        1   15.47624  855.85352   20.77288    1MHis
 24.174  1 MF        1   32.94461  742.38136   38.35686    Ctr
```

25.433	1	FM		1	23.48420	651.73194	24.00360	Arg
27.853	1	MF		1	85.03181	2646.11360	352.87555	bAla
28.589	1	FM		1	1562.33777	876.85865	2148.50414	Ala
32.185	1	MF	+	1	986.32849	1054.99395	1631.93636	Tau
35.276	1	MF		1	87.43316	1346.56510	184.64400	bABA
37.316	1	MF		1	9.10357	2114.21817	30.18516	GABA
42.515	1	FM		1	188.82249	620.29029	183.68783	Tyr
43.669	1	MF		1	25.21838	739.05841	29.22992	aABA
48.755	1	MM		1	138.24211	1432.91342	310.66475	EA
49.639	1	MM	+I	1	637.62939	1.00000	1.00000	dAVA
55.028	1	MM	+	1	317.56421	614.49249	306.04113	Val
56.587	1	MM		1	102.68363	646.82597	104.16465	Met
59.875	1	FM		1	9.29000	2405.52882	35.04759	Ctn
65.095	1	MF		1	36.39466	547.33623	31.24090	Trp
65.862	1	MF		1	206.74582	546.87627	177.31990	Ile
66.661	1	MF		1	216.28824	578.48967	196.22764	Phe
68.950	1	MF		1	142.56755	1664.93767	372.26340	Leu
69.919	2	MM		1	36.46005	934.34446	53.42641	HPro
74.884	1	FM		1	10.96073	3.62946e4	623.89670	Orn1
76.948	1	FM		1	13.88447	3.27395e4	712.90792	Lys1
78.607	1	FM		1	37.06162	1.12885e4	656.13424	Orn2
80.218	1	FM		1	31.30818	1.12918e4	554.43864	Lys2
81.129	2	FM		1	157.33038	1751.65986	432.20924	Pro

Totals without ISTD(s) : 3.09733e4

==

*** End of Report ***

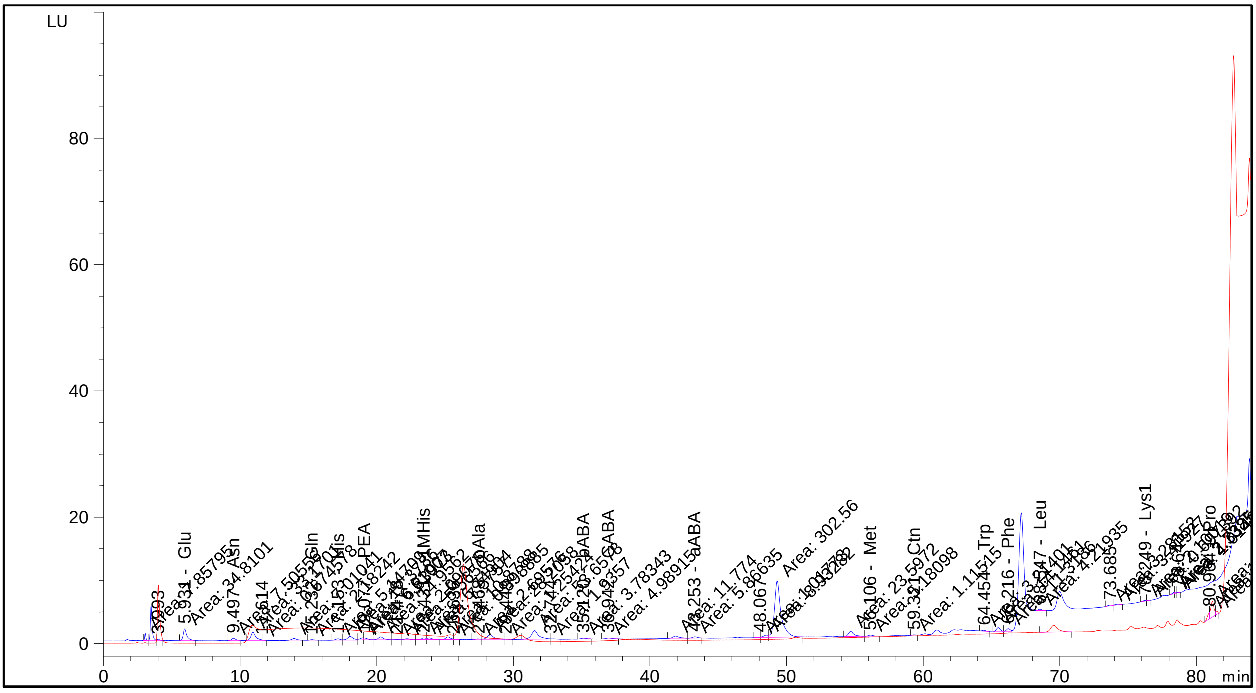

Figure S2. Chromatogram of free amino acids in rat liver lymphocytes.

==

Figure S2. Internal Standard Report

==

Sorted By : Retention Time
Multiplier: : 1.0000
Dilution: : 1.0000
Use Multiplier & Dilution Factor with ISTDs
Sample ISTD Information:

ISTD #	ISTD Amount [mcmol/l]	Name
1	1.00000	dAVA

Signal 1: FLD1 A, Ex=231, Em=445, TT

Signal 2: FLD1 B, Ex=231, Em=313, TT

RetTime [min]	Sig	Type	ISTD used	Area LU *s	Amt/Area ratio	Amount [mcmol/l]	Grp	Name
3.198	1		1	-	-	-		CA
3.476	1	VV +	1	49.18951	148.72575	24.17952		Asp
5.931	1	MM +	1	34.81008	107.97247	12.42245		Glu
9.497	1	MM	1	7.50556	113.80931	2.82325		Asn
10.924	1	MF +	1	33.17013	105.92805	11.61307		Ser
13.934	1	MM	1	7.01041	117.32935	2.71856		aAAA
15.232	1	MM	1	2.18242	149.81692	1.08066		Gln
17.153	1	MF	1	5.14709	211.71001	3.60158		His
18.048	1	MF	1	17.37864	198.94055	11.42689		Gly
18.935	1	MF	1	6.61007	113.13791	2.47174		3MHis
19.077	1	FM	1	6.83973	299.37662	6.76777		PEA
20.270	1	MF	1	14.95615	110.13666	5.44429		Thr
23.431	1	MF	1	6.74904	121.63327	2.71321		1MHis
23.851	1	FM	1	7.57570	99.71373	2.49670		Ctr
25.199	1	MF	1	10.87879	87.39461	3.14235		Arg
27.468	1	MF	1	2.69576	261.23287	2.32754		bAla
28.294	1	MF	1	28.20579	111.19383	10.36592		Ala
31.541	1	MF +	1	53.65783	113.16540	20.06947		Tau
35.120	1	MM	1	3.78343	143.92649	1.79976		bABA
36.942	1	MM	1	4.98915	183.26458	3.02200		GABA
41.871	1	MF	1	11.77401	83.19205	3.23739		Tyr
43.253	1	FM	1	5.86635	97.52980	1.89101		aABA
48.538	1	FM	1	8.93282	135.11367	3.98912		EA
49.305	1	MF +I	1	302.55963	1.00000	1.00000		dAVA
54.675	1	MF +	1	23.59722	85.41696	6.66184		Val
56.106	1	FM	1	5.18098	85.55173	1.46497		Met
59.321	1	MM	1	1.11515	1476.95625	5.44363		Ctn
64.454	1	MM	1	3.21401	69.33329	7.36509e-1		Trp
65.479	1	MF	1	14.14605	74.63528	3.48954		Ile
66.216	1	MF	1	11.31855	75.13354	2.81069		Phe
68.547	1	MM	1	4.21935	236.31174	3.29549		Leu
69.552	2	BBA	1	41.68841	244.33191	33.66546		HPro
74.274	1	FM	1	3.41927	3834.43345	43.33347		Orn1
76.249	1	MF	1	2.10019	2980.36420	20.68796		Lys1
78.423	1	FM	1	2.33258	3137.48431	24.18842		Orn2
80.063	1		1	-	-	-		Lys2
80.964	2	MF	1	12.08103	512.08160	20.44712		Pro

Totals without ISTD(s) : 305.82936

*** End of Report ***

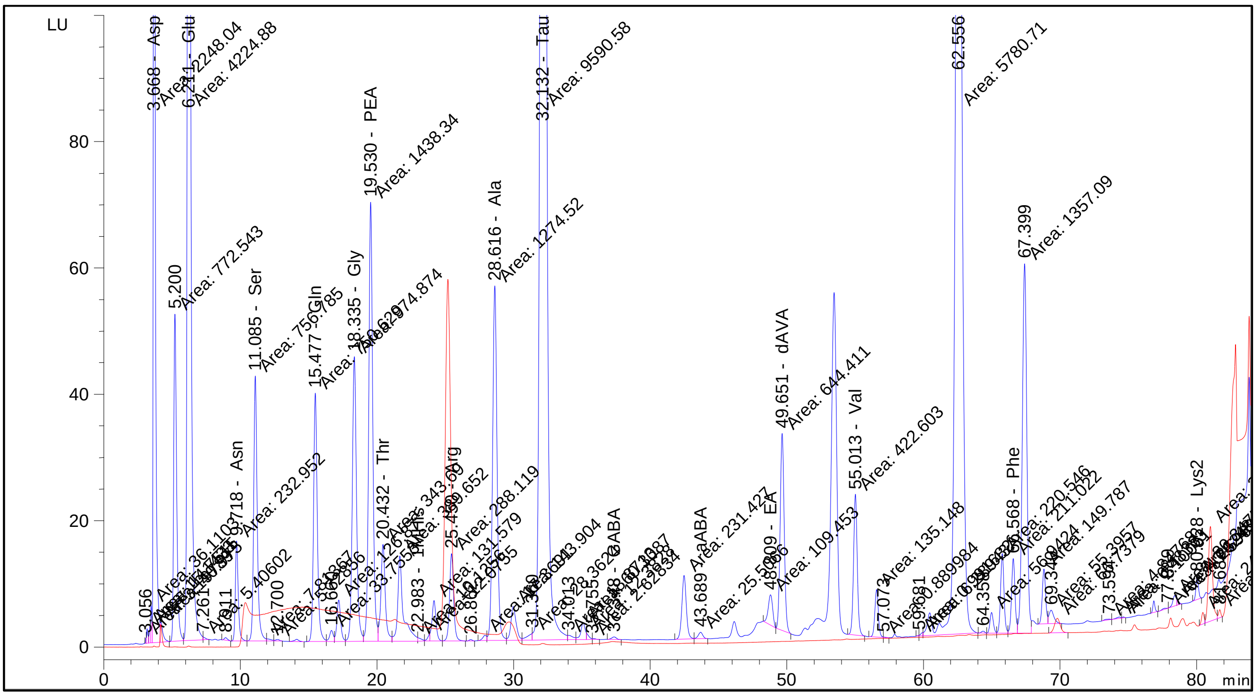

Figure S3. Chromatogram of free amino acids in rat spleen.

===

Figure S3. Internal Standard Report

===

Sorted By : Retention Time
Multiplier: : 1.0000
Dilution: : 1.0000
Use Multiplier & Dilution Factor with ISTDs
Sample ISTD Information:

ISTD #	ISTD Amount [nmol/g]	Name
1	1.00000	dAVA

Signal 1: FLD1 A, Ex=231, Em=445, TT
Signal 2: FLD1 B, Ex=231, Em=313, TT

RetTime [min]	Sig	Type	ISTD used	Area LU *s	Amt/Area ratio	Amount [nmol/g]	Grp	Name
3.285	1		1	-	-	-		CA
3.668	1	FM +	1	2248.03809	1012.06737	3530.61351		Asp
6.211	1	MF +	1	4224.88379	895.80883	5873.09731		Glu
9.718	1	MF	1	232.95193	905.80701	327.44555		Asn
11.085	1	FM +	1	756.78528	903.33880	1060.86575		Ser
14.120	1	BB	1	8.49233	950.73850	12.52925		aAAA
15.477	1	MF	1	750.62866	1084.70656	1263.49782		Gln
17.192	1	MF	1	126.87041	1645.88747	324.03923		His
18.335	1	MF	1	974.87390	2018.09888	3053.00823		Gly
18.811	1		1	-	-			3MHis
19.530	1	MF	1	1438.34082	4551.69952	1.01595e4		PEA
20.432	1	MF	1	343.68979	878.68585	468.63784		Thr
22.983	1	FM	1	10.12557	1006.88804	15.82114		1MHis
24.169	1	FM	1	131.57922	814.91270	166.39317		Ctr
25.450	1	FM	1	288.11908	720.11342	321.96600		Arg
27.896	1	MF	1	17.36915	3468.04077	93.47593		bAla
28.616	1	FM	1	1274.51721	956.60421	1891.97363		Ala
32.132	1	FM +	1	9590.58301	1124.17174	1.67307e4		Tau
35.329	1	FM	1	12.38279	1295.19377	24.88802		bABA
37.348	1	BB	1	10.03570	2044.71975	31.84334		GABA
42.471	1	MF	1	231.42690	669.98658	240.61185		Tyr
43.689	1	FM	1	25.50663	800.96307	31.70317		aABA
48.809	1	MM	1	109.45265	1520.36287	258.23234		EA

49.651	1	MM	+I	1	644.41095	1.00000	1.00000		dAVA
55.013	1	MF	+	1	422.60272	700.53068	459.40587		Val
56.563	1	MF		1	135.14841	713.84452	149.71029		Met
59.954	1	FM		1	6.28943	2349.10428	22.92717		Ctn
64.982	1	MF		1	56.94242	578.58225	51.12556		Trp
65.763	1	MF		1	220.54620	595.05138	203.65316		Ile
66.568	1	MF		1	211.02216	627.56609	205.50605		Phe
68.812	1	MF		1	149.78735	1894.70270	440.40609		Leu
69.794	2	MM		1	66.73795	819.37297	84.85776		HPro
74.323	1	FM		1	6.48333	3.04176e4	306.02682		Orn1
76.859	1	BB		1	19.60908	2.69297e4	819.45565		Lys1
78.384	1	MF		1	30.34751	1.16408e4	548.20285		Orn2
80.028	1	MM		1	48.01023	1.22046e4	909.27470		Lys2
80.998	2	MF		1	317.51379	1515.85672	746.89206		Pro

Totals without ISTD(s) : 5.08283e4

===

*** End of Report ***

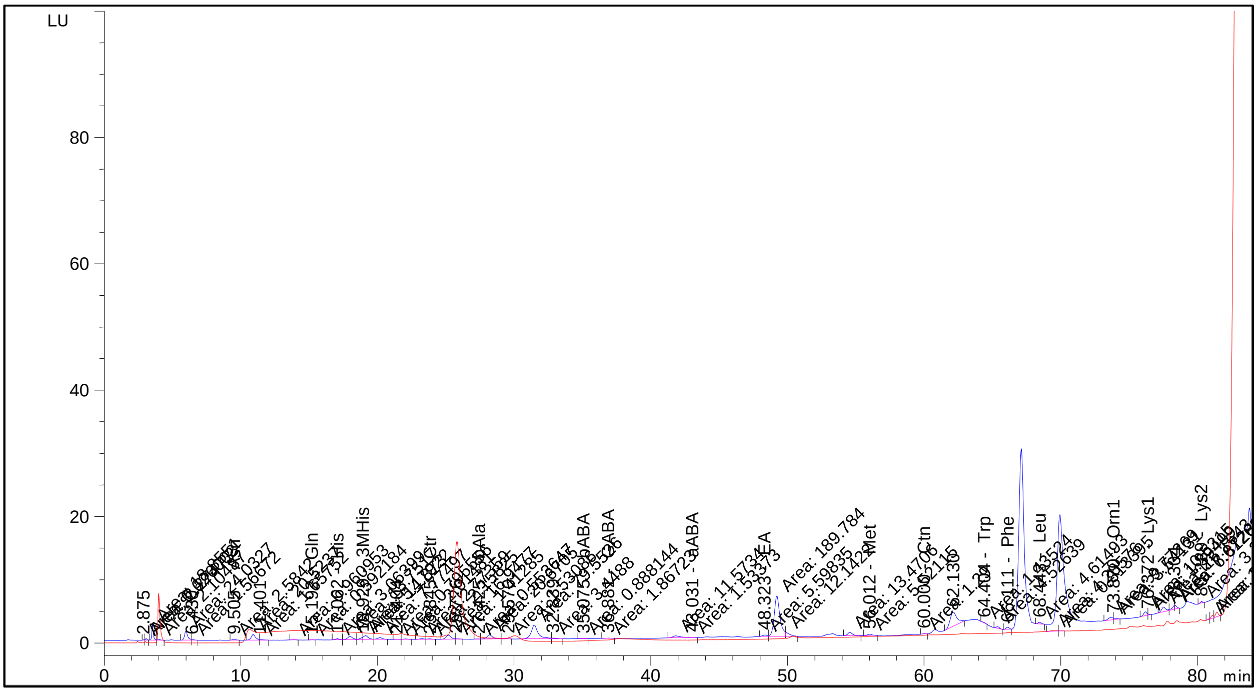

Figure S4. Chromatogram of free amino acids in rat spleen lymphocytes.

===

Figure S4. Internal Standard Report

===

Sorted By : Retention Time
Multiplier: : 1.0000
Dilution: : 1.0000
Use Multiplier & Dilution Factor with ISTDs
Sample ISTD Information:

ISTD #	ISTD Amount [mcmol/l]	Name
1	1.00000	dAVA

Signal 1: FLD1 A, Ex=231, Em=445, TT
Signal 2: FLD1 B, Ex=231, Em=313, TT

RetTime [min]	Sig	Type		ISTD used	Area LU *s	Amt/Area ratio	Amount [mcmol/l]	Grp	Name
3.232	1			1	-	-	-		CA
3.521	1	MF	+	1	18.85506	148.72575	14.77588		Asp
5.966	1	MF	+	1	24.03267	107.97247	13.67270		Glu
9.505	1	MM		1	2.58420	113.80931	1.54969		Asn

10.901	1	MF	+	1	20.52374	105.92805	11.45531	Ser
13.867	1	MM		1	9.60953e-1	117.32935	5.94084e-1	aAAA
15.192	1	MM		1	5.92184e-1	149.81692	4.67474e-1	Gln
17.062	1	MF		1	3.06399	211.71001	3.41797	His
17.969	1	MF		1	13.75173	198.94055	14.41517	Gly
18.929	1	MF		1	5.17922	113.13791	3.08754	3MHis
19.113	1	FM		1	14.87302	299.37662	23.46153	PEA
20.179	1	MF		1	7.77497	110.13666	4.51201	Thr
23.256	1	MF		1	2.77359	121.63327	1.77760	1MHis
23.845	1	MF		1	2.15825	99.71373	1.13396	Ctr
25.180	1	MF		1	16.94771	87.39461	7.80432	Arg
27.425	1	MF		1	5.53647e-1	261.23287	7.62079e-1	bAla
28.196	1	MF		1	20.67052	111.19383	12.11076	Ala
31.459	1	MF	+	1	79.55257	113.16540	47.43591	Tau
35.075	1	MM		1	8.88144e-1	143.92649	6.73540e-1	bABA
36.881	1	MM		1	1.86723	183.26458	1.80308	GABA
41.839	1	MF		1	11.57338	83.19205	5.07319	Tyr
43.031	1	FM		1	1.53373	97.52980	7.88180e-1	aABA
48.323	1	MF		1	5.59835	135.11367	3.98565	EA
49.217	1	MF	+I	1	189.78445	1.00000	1.00000	dAVA
54.564	1	MM	+	1	13.47065	85.41696	6.06278	Val
56.012	1	MM		1	5.52115	85.55173	2.48884	Met
60.000	1	MM		1	1.29000	1476.95625	10.03914	Ctn
64.404	1	MM		1	1.43524	69.33329	5.24331e-1	Trp
65.369	1	MM		1	4.52639	74.63528	1.78006	Ile
66.111	1	BB		1	5.50442	75.13354	2.17914	Phe
68.443	1	MM		1	4.61493	236.31174	5.74632	Leu
69.447	2	MF		1	4.28576	244.33191	5.51756	HPro
73.870	1	FM		1	3.54131	3834.43345	71.54911	Orn1
76.372	1	FM		1	1.39499	2980.36420	21.90690	Lys1
78.349	1	FM		1	6.81285	3137.48431	112.62889	Orn2
80.289	1	MM		1	3.29913	2013.14972	34.99569	Lys2
80.899	2	MF		1	1.46598	512.08160	3.95554	Pro

Totals without ISTD(s) : 454.13194

==

*** End of Report ***

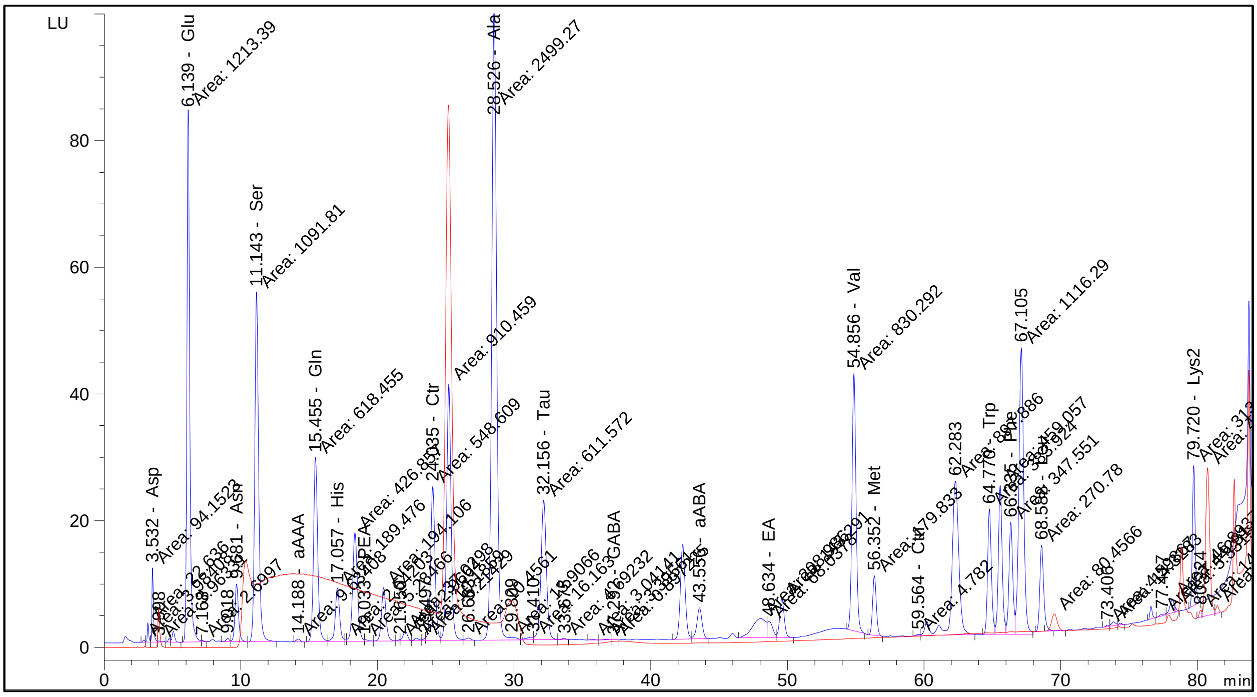

Figure S5. Chromatogram of free amino acids in rat plasma.

===

Figure S5.Internal Standard Report

===

Sorted By : Retention Time
Multiplier: : 1.0000
Dilution: : 1.0000
Use Multiplier & Dilution Factor with ISTDs
Sample ISTD Information:

ISTD #	ISTD Amount [mcmol/l]	Name
1	1.00000	dAVA

Signal 1: FLD1 A, Ex=231, Em=445, TT
Signal 2: FLD1 B, Ex=231, Em=313, TT

RetTime [min]	Sig	Type		ISTD used	Area LU *s	Amt/Area ratio	Amount [mcmol/l]	Grp	Name
3.103	1			1	-	-	-		CA
3.532	1	FM	+	1	94.15229	30.97527	20.35292		Asp
6.139	1	MF	+	1	1213.38989	27.38517	231.89786		Glu
9.681	1	VB		1	165.37498	34.03501	39.28046		Asn
11.143	1	MF	+	1	1091.81042	30.39083	231.56382		Ser
14.188	1	MF		1	9.63408	33.62352	2.26065		aAAA
15.455	1	MF		1	618.45526	154.89568	668.54162		Gln
17.057	1	MF		1	189.47554	59.21592	78.30194		His
18.340	1	MF		1	426.85718	73.11764	217.81391		Gly
18.511	1			1	-	-	-		3MHis
19.033	1	FM		1	5.28466	289.10521	10.66237		PEA
20.440	1	FM		1	194.10594	30.24409	40.96945		Thr
22.851	1	FM		1	10.18585	37.29586	2.65118		1MHis
24.035	1	FM		1	548.60944	28.71661	109.94548		Ctr
25.209	1	MF		1	910.45862	25.05550	159.20040		Arg
27.916	1			1	-	-	-		bAla
28.526	1	MF		1	2499.27393	32.87773	573.45135		Ala
32.156	1	MF	+	1	611.57178	42.51466	181.45420		Tau
35.731	1	MF		1	3.04141	65.16693	1.38319		bABA
37.292	1	FM		1	8.97225e-1	106.69165	6.68055e-1		GABA
42.328	1	BV		1	367.26764	23.23832	59.56187		Tyr
43.556	1	VB		1	131.35112	27.58057	25.28238		aABA
48.634	1	MF		1	68.03777	60.29279	28.62835		EA
49.591	1	FM	I	1	143.29108	1.00000	1.00000		dAVA
54.856	1	MM	+	1	830.29242	22.71269	131.60744		Val
56.352	1	MF		1	179.83292	24.17914	30.34526		Met
59.564	1	MF		1	4.78200	100.05841	3.33921		Ctn
64.770	1	MF		1	383.92435	19.12637	51.24588		Trp
65.558	1	FM		1	459.05673	20.43276	65.45973		Ile
66.335	1	FM		1	347.55130	21.91233	53.14818		Phe
68.588	1	FM		1	270.77988	64.67936	122.22582		Leu
69.517	2	MF		1	80.45660	46.65970	26.19898		HPro
74.366	1	VB		1	4.74321	2031.77850	67.25576		Orn1
76.594	1	BB		1	23.64708	2099.44116	346.46718		Lys1
78.152	1	MF		1	46.89372	324.00421	106.03425		Orn2
79.720	1	FM		1	313.24008	278.99337	609.89078		Lys2
80.732	2	MF		1	497.84137	83.84858	291.31815		Pro

Totals without ISTD(s) : 4588.40807

===

*** End of Report ***

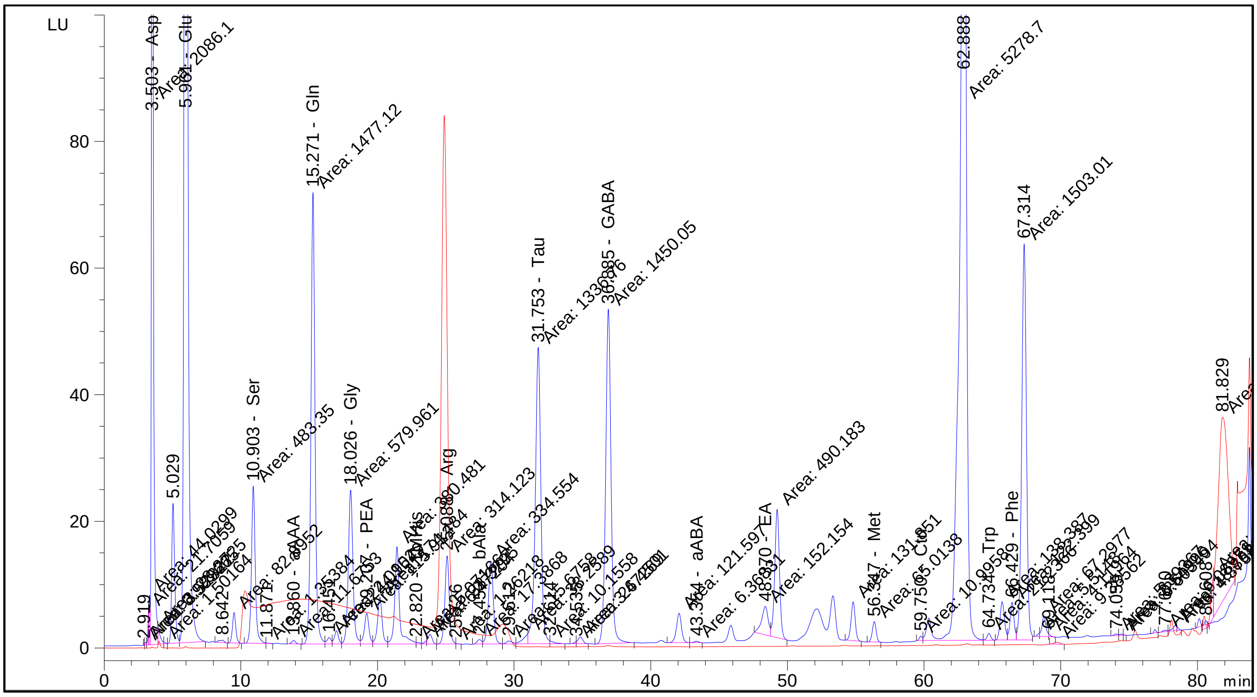

Figure S6. Chromatogram of free amino acids in rat hypothalamus.

===

Figure S6. Internal Standard Report

===

Sorted By : Retention Time

Multiplier: : 1.0000

Dilution: : 1.0000

Use Multiplier & Dilution Factor with ISTDs

Sample ISTD Information:

ISTD #	ISTD Amount [nmol/g]	Name
1	1.00000	dAVA

Signal 1: FLD1 A, Ex=231, Em=445, TT

Signal 2: FLD1 B, Ex=231, Em=313, TT

RetTime [min]	Sig	Type		ISTD used	Area LU *s	Amt/Area ratio	Amount [nmol/g]	Grp	Name
3.034	1	MF		1	3.52822	1163.33150	8.37338		CA
3.503	1	MF	+	1	2086.10400	1153.10579	4907.34459		Asp
5.961	1	VBA	+	1	5176.25830	883.33720	9327.90048		Glu
9.496	1	MF		1	82.89520	922.49866	156.00431		Asn
10.903	1	FM	+	1	483.35037	922.52164	909.66202		Ser
13.860	1	MM		1	11.67534	940.52357	22.40168		aAAA
15.271	1	MF		1	1477.12195	1201.47875	3620.54451		Gln
16.991	1	MF		1	44.15490	1605.98778	144.66469		His
18.026	1	MF		1	579.96112	2556.69672	3024.95933		Gly
18.635	1			1	-	-	-		3MHis
19.207	1	MF		1	115.91191	7181.86501	1698.27004		PEA
20.169	1	MF		1	174.48395	883.59713	314.52214		Thr
22.820	1	MF		1	5.07166	1186.76534	12.27881		1MHis
23.847	1	FM		1	32.32951	796.38175	52.52450		Ctr
25.088	1	MF		1	314.12268	691.31064	443.01046		Arg
27.453	1	MF		1	17.38676	3798.60491	134.73616		bAla
28.294	1	MF		1	334.55423	983.08216	670.96178		Ala
31.753	1	MF	+	1	1336.76147	1261.46267	3440.08971		Tau
34.830	1	FM		1	26.45014	1487.96568	80.29016		bABA
36.885	1	MM		1	1450.04944	2466.57268	7296.56023		GABA
42.062	1	MF		1	121.59731	655.97820	162.72519		Tyr

```
 43.354  1 FM        1    6.36831  814.25396   10.57853    aABA
 48.370  1 MF        1  152.15448 1501.10341  465.94730    EA
 49.238  1 FM  +I    1  490.18335    1.00000    1.00000    dAVA
 54.798  1 MM  +     1  131.85081  668.78875  179.89257    Val
 56.347  1 MM        1   65.01379  698.64020   92.66175    Met
 59.750  1 MF        1   10.99579 2671.06069   59.91722    Ctn
 64.734  1 FM        1   21.23659  559.51974   24.24051    Trp
 65.702  1 MF        1  138.38708  606.73733  171.29225    Ile
 66.429  1 MF        1  122.39880  633.55684  158.19917    Phe
 68.755  1 MF        1   67.29769 1828.91147  251.09282    Leu
 69.725  2 MM        1    9.38562 1073.31530   20.55093    HPro
 74.567  1 FM        1 6.03494e-1 2.96364e4    36.48717    Orn1
 76.886  1 BB        1    6.24781 2.76980e4   353.03464    Lys1
 78.501  1 FM        1    4.37380 9345.61092   83.38886    Orn2
 80.124  1 BB        1   16.87332 6902.52791  237.60206    Lys2
 81.191  2 MF        1   90.47009 1794.61328  331.22061    Pro
Totals without ISTD(s) :                3.89039e4
=======================================================================
                         *** End of Report ***
```

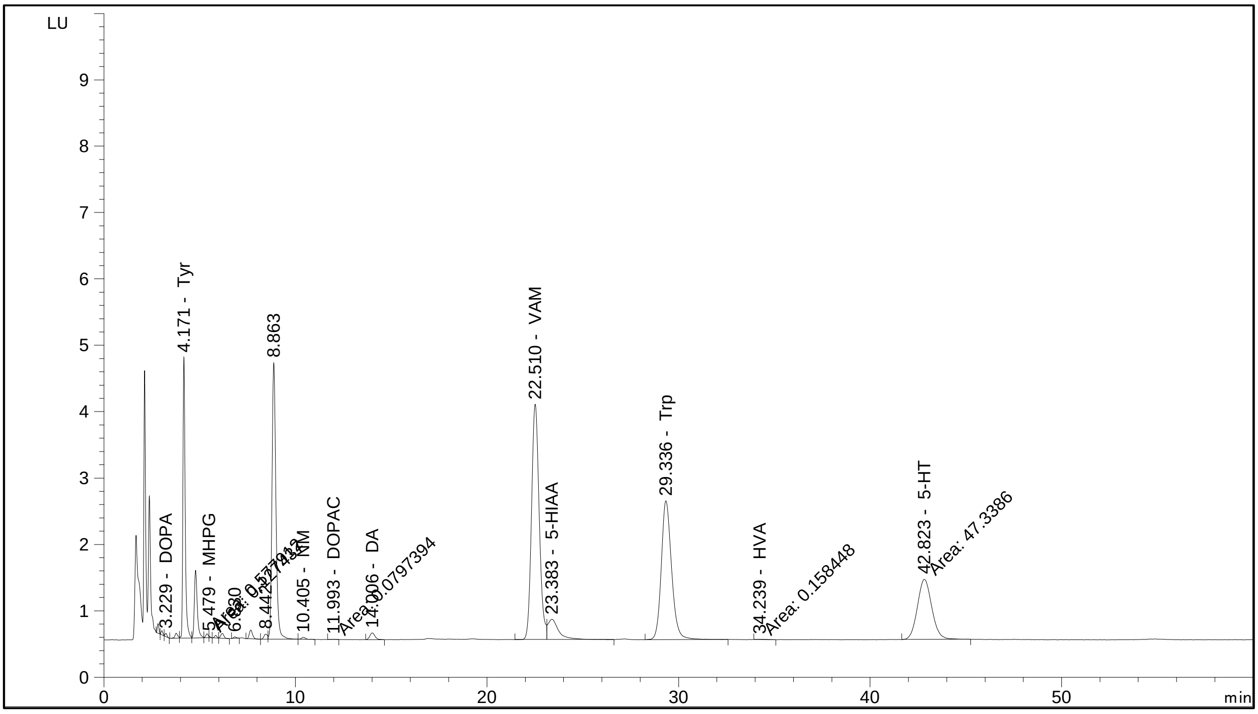

Figure S7. Chromatogram of biogenic amines in rat liver.

Note: in this and Supplementary Figures 7-12, 14. – 3-MT, 3-methoxytyramine; 5-HIAA, 5-hydroxyindoleacetic acid; 5-HT, serotonin; 5-HTP, 5-hydroxytryptophan; DA, dopamine; DOPA, dioxyphenylalanine; DOPAC, dioxyphenylacetic acid; E, epinephrine; HVA, homovanillic acid; MHPG, 3-methoxy,4-hydroxyphenyl glycol; NE, norepinephrine; NM, normetanephrine; Sal, salsolinol; Trp, tryptophan; Tyr, tyrosine.

```
=======================================================================
                Figure S7. Internal Standard Report
=======================================================================
Sorted By             :      Retention Time
Multiplier:                   :      1.0000
Dilution:                     :      1.0000
Use Multiplier & Dilution Factor with ISTDs
Sample ISTD Information:
ISTD  ISTD Amount   Name
  #    [nmol/g]
----|-------------|-------------------------
  1      1.00000   VAM
```

Signal 1: FLD1 A, Ex=280, Em=340

RetTime [min]	Sig	Type	ISTD used	Area LU *s	Amt/Area ratio	Amount [nmol/g]	Grp	Name
3.229	1	VB	1	3.71576e-1	186.35902	6.98365e-1		DOPA
4.171	1	VV	1	34.88512	112.39301	39.54252		Tyr
4.782	1	VV	1	10.88908	184.65148	20.27818		NE
5.479	1	FM	1	1.27437e-1	3402.04777	4.37240		MHPG
6.633	1		1	-	-	-		E
7.662	1	BB	1	1.62995	12.78420	2.10151e-1		5-HTP
10.405	1	BB	1	4.81161e-1	57.97828	2.81346e-1		NM
11.993	1	MM	1	7.97394e-2	1353.03107	1.08809		DOPAC
14.006	1	BB	1	1.97590	170.83753	3.40434		DA
17.654	1		1	-	-	-		Sal
22.510	1	BV I	1	99.15511	1.00000	1.00000		VAM
23.383	1	VB	1	12.31334	18.98405	2.35749		5-HIAA
26.001	1		1	-	-	-		VA
29.336	1	BB	1	78.42071	14.43851	11.41926		Trp
34.239	1	MM	1	1.58448e-1	466.54696	7.45535e-1		HVA
41.665	1		1	-	-	-		3-MT
42.823	1	MM	1	47.33860	25.41625	12.13422		5-HT

Totals without ISTD(s) : 96.53190

*** End of Report ***

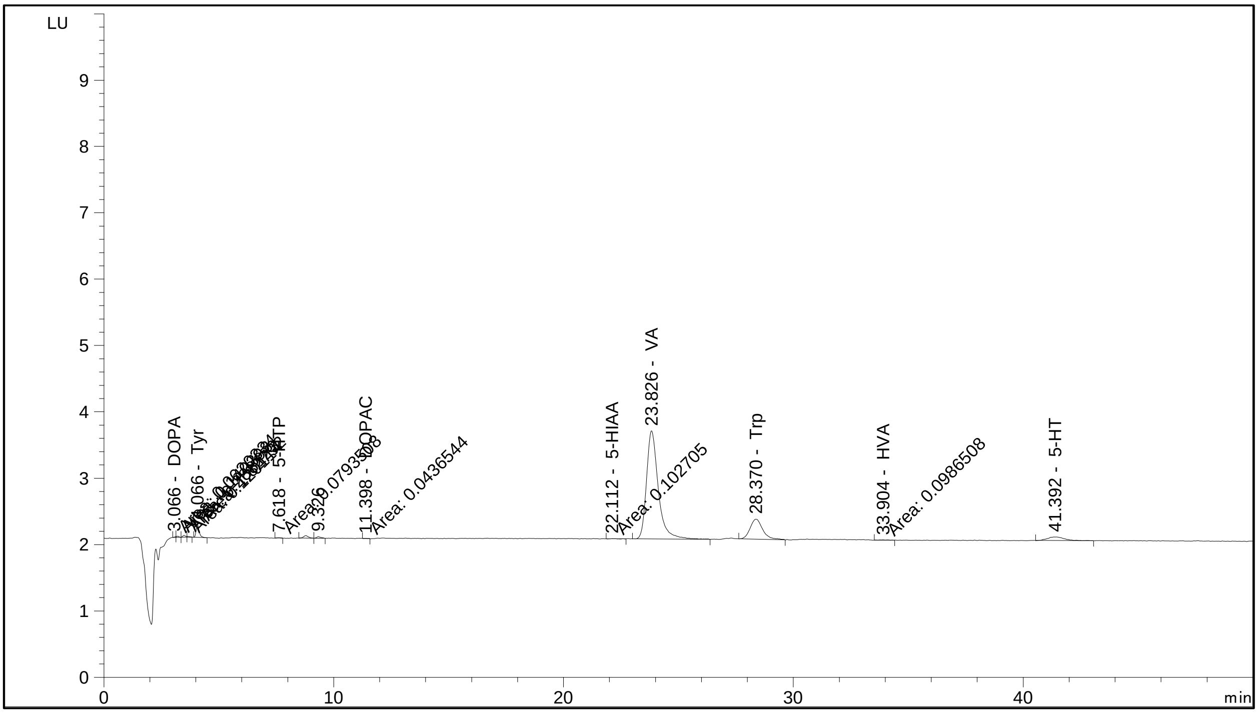

Figure S8. Chromatogram of biogenic amines in rat liver lymphocytes.

Figure S8. Internal Standard Report

Sorted By : Retention Time
Multiplier: : 1.0000
Dilution: : 1.0000
Use Multiplier & Dilution Factor with ISTDs
Sample ISTD Information:

ISTD #	ISTD Amount [mcM]	Name
1	1.00000	VA

Signal 1: FLD1 A, Ex=280, Em=340

RetTime [min]	Sig	Type	ISTD used	Area LU *s	Amt/Area ratio	Amount [mcM]	Grp	Name
3.066	1	MF	1	6.20334e-2	8.79515	9.38627e-3		DOPA
4.066	1	MM	1	1.82792	16.50855	5.19148e-1		Tyr
4.555	1		1	-	-	-		NE
5.105	1		1	-	-	-		MHPG
6.476	1		1	-	-	-		E
7.618	1	MM	1	7.93508e-2	6.11956e-1	8.35403e-4		5-HTP
9.818	1		1	-	-	-		NM
11.398	1	MM	1	4.36544e-2	111.02275	8.33805e-2		DOPAC
13.299	1		1	-	-	-		DA
16.647	1		1	-	-	-		Sal
22.112	1	MM	1	1.02705e-1	5.47939	9.68161e-3		5-HIAA
23.826	1	BB I	1	58.12669	1.00000	1.00000		VA
28.370	1	BB +	1	11.66432	1.97464	3.96252e-1		Trp
33.904	1	MM	1	9.86508e-2	44.00311	7.46807e-2		HVA
38.159	1		1	-	-	-		3-MT
41.392	1	BB	1	3.04401	6.22915e-1	3.26212e-2		5-HT

Totals without ISTD(s) : 1.12598

*** End of Report ***

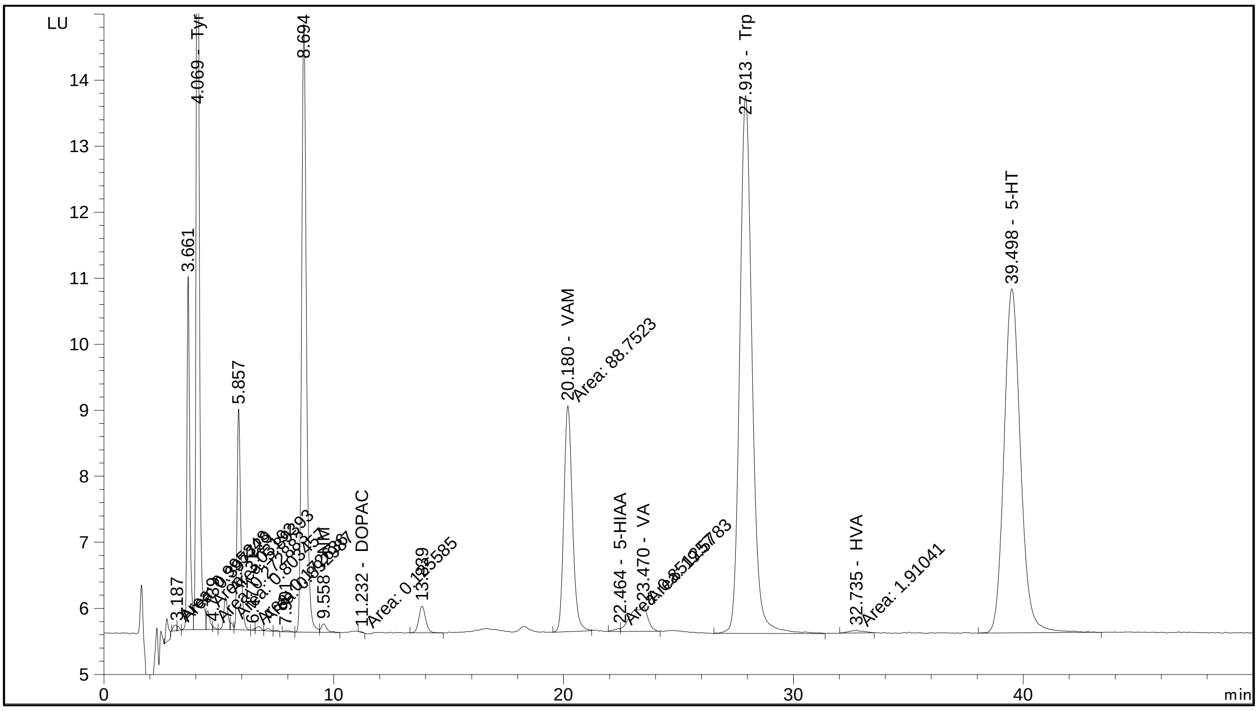

Figure S9. Chromatogram of biogenic amines in rat spleen.

Figure S9. Internal Standard Report

Sorted By : Retention Time
Multiplier: : 1.0000
Dilution: : 1.0000
Use Multiplier & Dilution Factor with ISTDs
Sample ISTD Information:

ISTD #	ISTD Amount [nmol/g]	Name
1	1.00000	VAM

Signal 1: FLD1 A, Ex=280, Em=340

RetTime [min]	Sig	Type	ISTD used	Area LU *s	Amt/Area ratio	Amount [nmol/g]	Grp	Name

RetTime [min]	Sig	Type	ISTD used	Area LU *s	Amt/Area ratio	Amount [mcM]	Grp	Name
3.082	1	MF	1	9.95348e-1	186.35902	2.09000		DOPA
4.069	1	VV	1	134.62944	112.39301	170.49037		Tyr
4.515	1	MF	1	3.03192	184.65148	6.30800		NE
5.065	1		1	-	-	-		MHPG
6.477	1	MF	1	1.72636e-1	104.09140	2.02473e-1		E
7.564	1	VB	1	2.07990e-1	12.78420	2.99597e-2		5-HTP
9.558	1	VB	1	2.32668	57.97828	1.51993		NM
11.232	1	MM	1	1.25585e-1	1353.03107	1.91454		DOPAC
12.813	1		1	-	-	-		DA
15.994	1		1	-	-	-		Sal
20.180	1	MM I	1	88.75227	1.00000	1.00000		VAM
22.464	1	MF	1	8.51257e-1	18.98405	1.82083e-1		5-HIAA
23.470	1	FM	1	19.57829	6.99353	1.54274		VA
27.913	1	BB	1	305.72672	14.43851	49.73661		Trp
32.735	1	MM	1	1.91041	466.54696	10.04251		HVA
36.440	1		1	-	-	-		3-MT
39.498	1	BB	1	262.08621	25.41625	75.05441		5-HT

Totals without ISTD(s) : 319.11362

*** End of Report ***

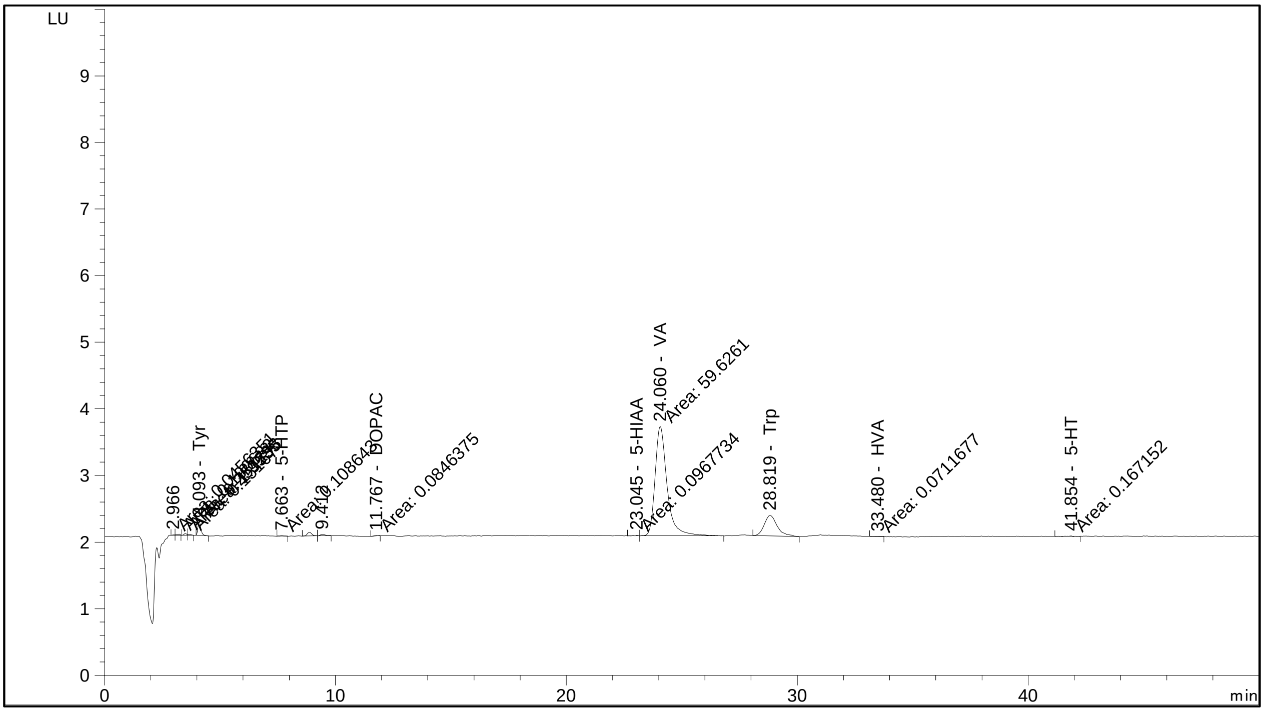

Figure S10. Chromatogram of biogenic amines in rat spleen lymphocytes.

Figure S10. Internal Standard Report

Sorted By : Retention Time
Multiplier: : 1.0000
Dilution: : 1.0000
Use Multiplier & Dilution Factor with ISTDs
Sample ISTD Information:

ISTD #	ISTD Amount [mcM]	Name
1	1.00000	VA

Signal 1: FLD1 A, Ex=280, Em=340

RetTime [min]	Sig	Type	ISTD used	Area LU *s	Amt/Area ratio	Amount [mcM]	Grp	Name

3.066	1	MF	1	6.20334e-2	8.79515	9.38627e-3		DOPA
4.066	1	MM	1	1.82792	16.50855	5.19148e-1		Tyr
4.555	1		1	-	-	-		NE
5.105	1		1	-	-	-		MHPG
6.476	1		1	-	-	-		E
7.618	1	MM	1	7.93508e-2	6.11956e-1	8.35403e-4		5-HTP
9.818	1		1	-	-	-		NM
11.398	1	MM	1	4.36544e-2	111.02275	8.33805e-2		DOPAC
13.299	1		1	-	-	-		DA
16.647	1		1	-	-	-		Sal
22.112	1	MM	1	1.02705e-1	5.47939	9.68161e-3		5-HIAA
23.826	1	BB I	1	58.12669	1.00000	1.00000		VA
28.370	1	BB +	1	11.66432	1.97464	3.96252e-1		Trp
33.904	1	MM	1	9.86508e-2	44.00311	7.46807e-2		HVA
38.159	1		1	-	-	-		3-MT
41.392	1	BB	1	3.04401	6.22915e-1	3.26212e-2		5-HT

Totals without ISTD(s) : 1.12598

==

*** End of Report ***

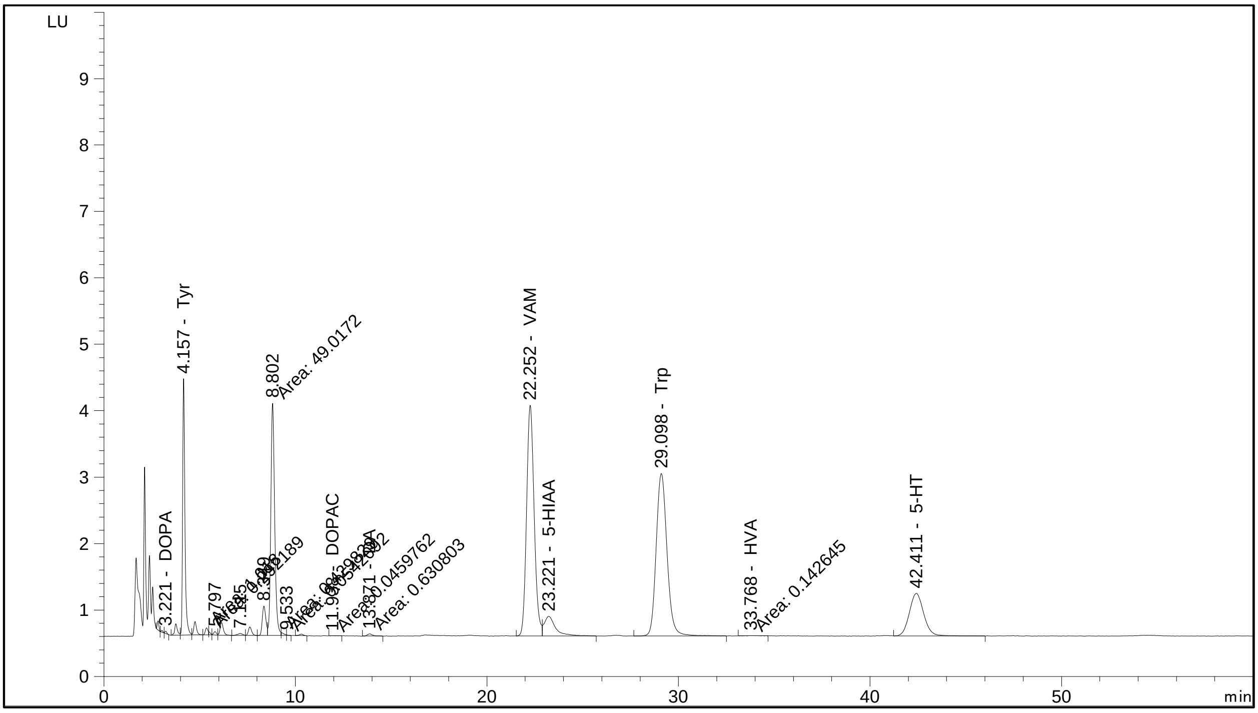

Figure S11. Chromatogram of biogenic amines in rat hypothalamus.

==

Figure S11. Internal Standard Report

==

Sorted By : Retention Time
Multiplier: : 1.0000
Dilution: : 1.0000
Use Multiplier & Dilution Factor with ISTDs
Sample ISTD Information:

ISTD #	ISTD Amount [nmol/g]	Name
1	1.00000	VAM

Signal 1: FLD1 A, Ex=280, Em=340

RetTime [min]	Sig	Type	ISTD used	Area LU *s	Amt/Area ratio	Amount [nmol/g]	Grp	Name
3.221	1	VB	1	2.54122e-1	186.35902	4.93968e-1		DOPA
4.157	1	VV	1	31.81875	112.39301	37.30177		Tyr

4.752	1	VB		1	2.06009	184.65148	3.96777	NE
5.354	1	MF		1	1.01800	3402.04777	36.12390	MHPG
6.766	1			1	-	-	-	E
7.615	1	VB		1	1.54704	12.78420	2.06292e-1	5-HTP
10.309	1	BB		1	3.61797e-1	57.97828	2.18795e-1	NM
11.938	1	MM		1	4.59762e-2	1353.03107	6.48855e-1	DOPAC
13.871	1	MM		1	6.30803e-1	170.83753	1.12405	DA
17.295	1			1	-	-	-	Sal
22.252	1	BV	I	1	95.87227	1.00000	1.00000	VAM
23.221	1	VB		1	12.32977	18.98405	2.44147	5-HIAA
26.001	1			1	-	-	-	VA
29.098	1	BB		1	96.57403	14.43851	14.54419	Trp
33.768	1	MM		1	1.42645e-1	466.54696	6.94160e-1	HVA
40.596	1			1	-	-	-	3-MT
42.411	1	BB		1	33.68652	25.41625	8.93048	5-HT

Totals without ISTD(s) : 106.69569

==

*** End of Report ***

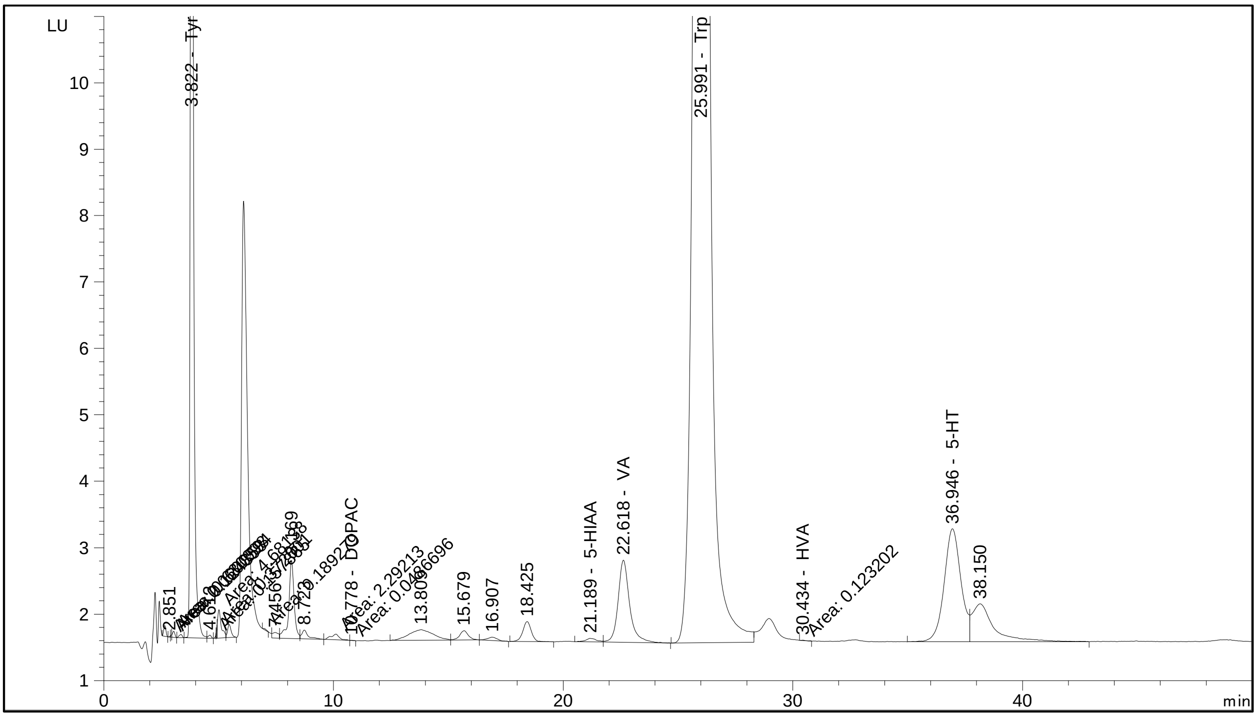

Figure S12. Chromatogram of biogenic amines in rat plasma.

==

Figure S12. Internal Standard Report

==

Sorted By : Retention Time
Multiplier: : 1.0000
Dilution: : 1.0000
Use Multiplier & Dilution Factor with ISTDs
Sample ISTD Information:

ISTD #	ISTD Amount [mcM]	Name
1	1.00000	VA

Signal 1: FLD1 A, Ex=280, Em=340

RetTime [min]	Sig	Type	ISTD used	Area LU *s	Amt/Area ratio	Amount [mcM]	Grp	Name
2.945	1	FM	1	1.24858e-1	8.79515	2.53269e-2		DOPA
3.822	1	BV +	1	180.83676	16.50855	68.85235		Tyr
4.452	1		1	-	-	-		NE

4.908	1	MF		1	3.72001e-1	9.70569	8.32708e-2	MHPG
6.149	1			1	-	-	-	E
6.998	1	MM		1	1.89279e-1	6.11956e-1	2.67144e-3	5-HTP
9.182	1			1	-	-	-	NM
10.778	1	FM		1	4.86696e-2	111.02275	1.24622e-1	DOPAC
12.111	1			1	-	-	-	DA
14.940	1			1	-	-	-	Sal
21.189	1	BV		1	1.89880	5.47939	2.39957e-1	5-HIAA
22.618	1	VB	I	1	43.35875	1.00000	1.00000	VA
25.991	1	BV	+	1	1387.54565	1.97464	63.19138	Trp
30.434	1	MM		1	1.23202e-1	44.00311	1.25033e-1	HVA
33.472	1			1	-	-	-	3-MT
36.946	1	BV		1	87.10741	6.22915e-1	1.25143	5-HT

Totals without ISTD(s) : 133.89605

===

*** End of Report ***

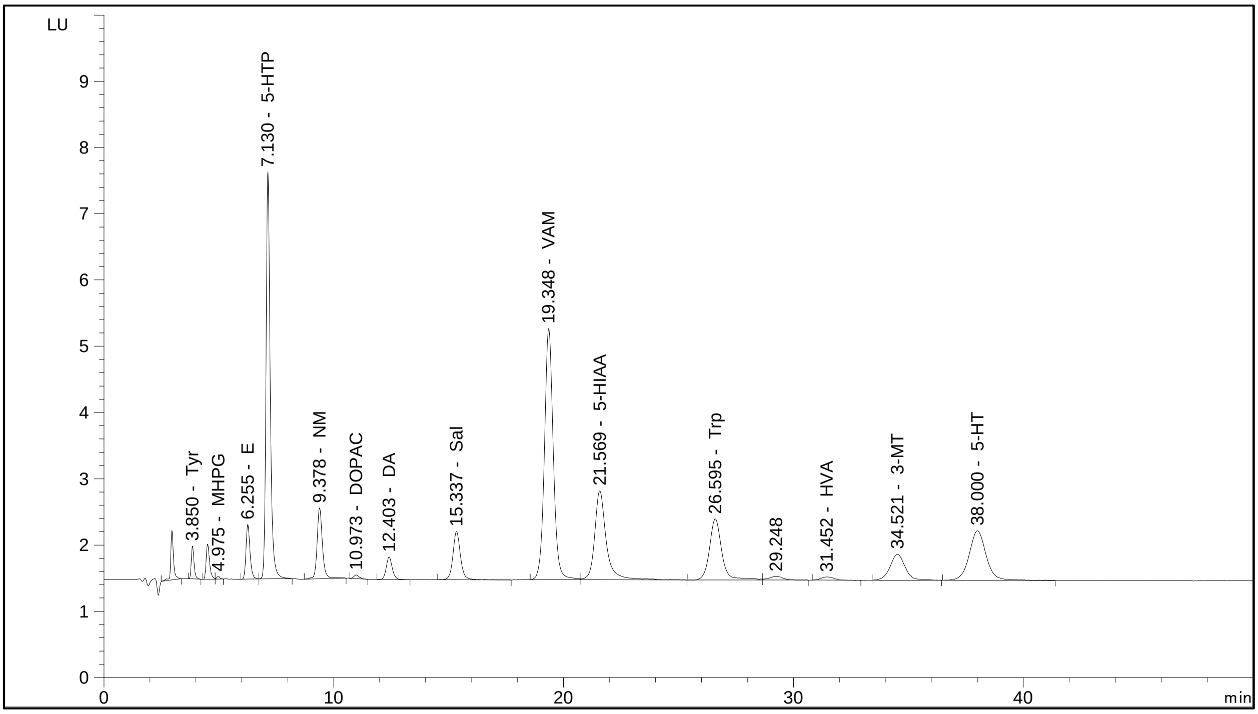

Figure S13. Typical chromatogram of a mixture of biogenic amine standards.

===

Figure S13. Internal Standard Report

===

Sorted By : Retention Time
Multiplier: : 1.0000
Dilution: : 1.0000
Use Multiplier & Dilution Factor with ISTDs
Sample ISTD Information:

ISTD #	ISTD Amount [nmol/g]	Name
1	1.00000	VAM

Signal 1: FLD1 A, Ex=280, Em=340

RetTime [min]	Sig	Type	ISTD used	Area LU *s	Amt/Area ratio	Amount [nmol/g]	Grp	Name
2.956	1	BB	1	6.41091	186.35902	11.77477		DOPA
3.850	1	BB	1	4.14451	112.39301	4.59087		Tyr
4.508	1	BV	1	5.57525	184.65148	10.14611		NE
4.975	1	VB	1	4.17296e-1	3402.04777	13.99157		MHPG
6.255	1	BV	1	10.12136	104.09140	10.38330		E

7.130	1	VB		1	77.95903	12.78420	9.82250		5-HTP
9.378	1	BB		1	17.54685	57.97828	10.02643		NM
10.973	1	BB		1	1.01678	1353.03107	13.55867		DOPAC
12.403	1	BB		1	6.45315	170.83753	10.86518		DA
15.337	1	BB		1	16.92297	65.58875	10.93926		Sal
19.348	1	BV	I	1	101.46545	1.00000	1.00000		VAM
21.569	1	VB		1	47.90625	18.98405	8.96319		5-HIAA
22.859	1			1	-	-	-		VA
26.595	1	BV		1	34.94226	14.43851	4.97228		Trp
31.452	1	BB		1	2.21720	466.54696	10.19489		HVA
34.521	1	BB		1	18.21004	58.00635	10.41042		3-MT
38.000	1	BB		1	38.66373	25.41625	9.68494		5-HT

Totals without ISTD(s) : 150.32436

==

*** End of Report ***

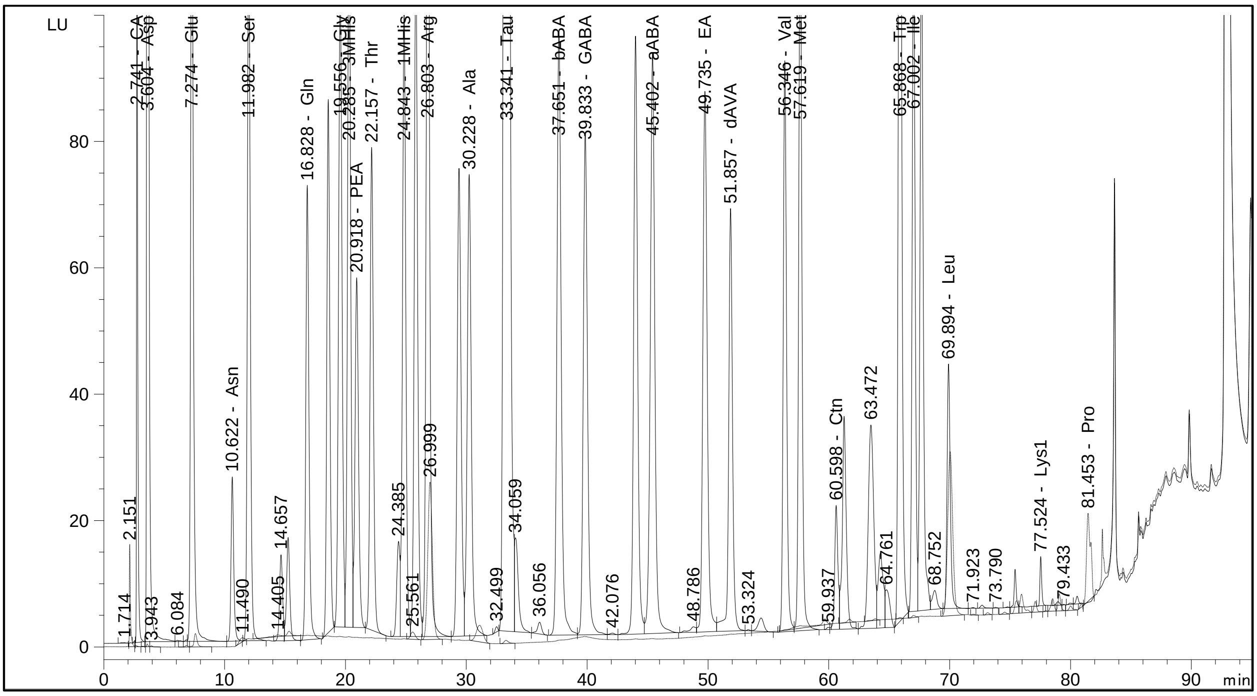

Figure S14. Typical chromatogram of a mixture of amino acids standards.

==

Figure S14. Internal Standard Report

==

Sorted By : Retention Time
Multiplier: : 1.0000
Dilution: : 1.0000
Use Multiplier & Dilution Factor with ISTDs
Sample ISTD Information:

ISTD #	ISTD Amount [nmol/g]	Name
1	1.00000	dAVA

Signal 1: FLD1 A, Ex=231, Em=445, TT
Signal 2: FLD1 B, Ex=231, Em=313, TT

RetTime [min]	Sig	Type		ISTD used	Area LU *s	Amt/Area ratio	Amount [nmol/g]	Grp	Name
2.741	1	VB		1	778.64374	3476.20196	1831.14635		CA
3.604	1	BV	+	1	4418.45752	2975.72004	8894.91937		Asp
7.274	1	VB		1	2133.28491	1658.16462	2393.07202		Glu
10.622	1	BB		1	402.02353	1341.22888	364.78219		Asn
11.982	1	BB	+	1	1959.09106	1183.79875	1568.95961		Ser

16.687	1			1	-	-	-	aAAA
16.828	1	BB		1	1299.35620	1464.27649	1287.15419	Gln
18.565	1	BB		1	1465.04285	1.21886e4	1.20805e4	His
19.556	1	BV		1	2051.61475	1300.47002	1804.99257	Gly
20.285	1	VV		1	2138.88477	1257.95084	1820.24697	3MHis
20.918	1	VB		1	1128.63086	7703.80533	5882.15524	PEA
22.157	1	BB		1	1473.76257	5201.27789	5185.81289	Thr
24.843	1	VV		1	1918.97559	1457.05571	1891.58068	1MHis
25.812	1	VV		1	2133.91309	1083.85992	1564.69301	Ctr
26.803	1	VB		1	2947.51245	781.90999	1559.16355	Arg
29.387	1	BV		1	1666.33582	2010.93609	2266.94017	bAla
30.228	1	VB		1	1716.24927	1136.40037	1319.44410	Ala
33.341	1	BV	+	1	1.47960e4	918.89679	9197.93587	Tau
37.651	1	BV		1	2356.56055	1153.47106	1838.92732	bABA
39.833	1	VV		1	1959.07776	1782.45320	2362.37627	GABA
43.994	1	BV		1	2172.45532	882.03720	1296.33431	Tyr
45.402	1	VB		1	2423.66821	962.12508	1577.55302	aABA
49.735	1	VV		1	2144.83472	1355.72079	1967.17660	EA
51.857	1	VB	+I	1	1478.15759	1.00000	1.00000	dAVA
56.346	1	BV		1	1744.16138	1170.57426	1381.22648	Val
57.619	1	VB		1	2157.39355	806.38206	1176.92692	Met
60.598	1	VV		1	369.25052	6146.13388	1535.33232	Ctn
65.868	1	BB		1	3431.82202	665.30806	1544.63832	Trp
67.002	1	BV		1	1856.31506	858.64381	1078.31090	Ile
67.657	1	VV		1	2039.28186	1018.64057	1405.32732	Phe
69.894	1	BB		1	720.50995	2585.17765	1260.11342	Leu
70.037	2	BB		1	764.96747	4727.83351	2446.72073	HPro
75.404	1	VB		1	70.46190	5.03094e4	2398.18611	Orn1
77.524	1	BB	+	1	79.70206	3.58794e4	1934.61478	Lys1
78.955	1	BV		1	11.57525	3.49847e4	273.96071	Orn2
80.538	1	VB		1	18.50900	1.29831e4	162.57006	Lys2
81.453	2	BB		1	330.63034	6655.36982	1488.65534	Pro

Totals without ISTD(s) : 8.80424e4

==

*** End of Report ***
